# Chelating silica nanoparticles for efficient antibiotic delivery and particle imaging in Gram-negative bacteria†

**DOI:** 10.1039/d2na00884j

**Published:** 2023-02-20

**Authors:** Asier R. Muguruza, Alessandro di Maio, Nikolas J. Hodges, Jessica M. A. Blair, Zoe Pikramenou

**Affiliations:** a School of Chemistry, College of Engineering and Physical Sciences, University of Birmingham Edgbaston B15 2TT UK z.pikramenou@bham.ac.uk +44 (0)121 4142290; b Birmingham Advanced Light Microscopy Facility, University of Birmingham Edgbaston B15 2TT UK; c School of Biosciences, University of Birmingham Edgbaston B15 2TT UK; d Institute of Microbiology and Infection, College of Medical and Dental Sciences, University of Birmingham Edgbaston B15 2TT UK J.M.A.Blair@bham.ac.uk +44 (0)121 4147606

## Abstract

The inefficacy of antibiotics against Gram-negative bacteria is a major challenge for treatment of many clinically important bacterial infections. The complex structure of the double cell membrane of Gram-negative bacteria makes it inaccessible to many key antibiotics such as vancomycin and also presents a major challenge for drug development. In this study we design of a novel hybrid silica nanoparticle system bearing membrane targeting groups with the antibiotic encapsulated together with a ruthenium luminescent tracking agent, for optical detection of the nanoparticle delivery in the bacterial cell. The hybrid system shows delivery of vancomycin and efficacy against a library of Gram negative bacterial species. Evidence of penetration of nanoparticles in bacteria cells is achieved *via* luminescence of the ruthenium signal. Our studies show that nanoparticles modified with aminopolycarboxylate chelating groups are an effective delivery system in bacterial growth inhibition in species whereas the molecular antibiotic is ineffective. This design provides a new platform for delivery of antibiotics that cannot alone penetrate the bacterial membrane.

## Abstract 2

Silica nanoparticles decorated with aminocarboxylate ligands are novel vehicles to deliver antibiotics which are otherwise not able to penetrate the bacteria membrane and can also include agents for tracking and imaging.

## Graphical abstract

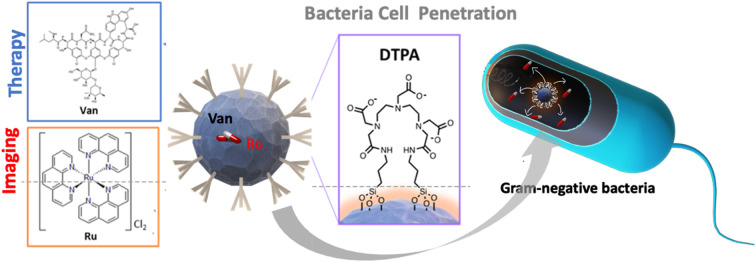

## Introduction

Gram-negative bacterial species, including *E. coli*, are major causes of infectious diseases worldwide and account for all organisms in the “critical” category of the WHO list of antibiotic-resistant “priority pathogens”.^1^ These bacteria are intrinsically drug-resistant due to the presence of an impermeable outer membrane.^2,3^ The design of novel antibiotics with activity against Gram-negative bacteria can be challenging due to the drug's size and hydrophobicity properties in order for the drug to cross the membrane and access intracellular targets. Nanoscale materials are attractive vehicles for the delivery of therapeutic agents with wide chemical versatility which provides potential to overcome multi-drug resistance mechanisms.^4^

Silica nanoparticles are attractive vehicles due to their excellent biocompatibility, facile synthesis with tunability of their size and surface chemistry for functionalisation. The most popular approach using silica nanoparticles for drug delivery is the formation of mesoporous particles. Indeed, such approach leads to high loading capacity with a plethora of agents, from nucleic acids to cancer drugs and several antibiotics.^5–7^ However, there are two drawbacks which limit their translation to the clinic: the porosity of the particle is induced by the use of a surfactant such as cetyl trimethyl bromide which is cytotoxic and needs to be fully removed from the particle;^6^ secondly, the high porosity leads to uncontrolled, “burst” release of the adsorbed agent in fluid delivery conditions which limits their application in targeted delivery. Approaches to limit the leakage of the drug usually involve functionalization of the particle's outer surface with a range of macromolecular structures to block the pores such as polymers,^8,9^ peptides^10^ or lipids.^11^

To tackle the challenge of both membrane permeation and controlled antibiotic delivery we chose to develop a hybrid, non-mesoporous silica nanoparticle system with surface functionalization to enhance penetration of the bacterial cell and inclusion of a metal complex to allow optical tracking of the nanoparticle's fate inside the cell. Hybrid nanoparticles have been previously developed with designs involving covalent modification of particle surface with organic fluorophores,^12^ or condensation of silyl-modified organic fluorophores in the silica network^13^ or by covalently linking quantum dots to the silica framework of porous nanoparticles.^14^ We have previously explored tracking of nanoparticles using metal complexes as photostable luminescent agents with characteristic emission properties which offer optical imaging modalities based on their distinct signal, away from any scattering, with long luminescent lifetimes, to probe their localisation in tissues,^15,16^ under flow conditions^17,18^ and in drug delivery.^19^ In this study we chose to use ruthenium tris phenanthroline, Ru, as a luminescent photostable probe which is much more resistant to photobleaching than organic dyes. In addition, its encapsulation in the silica framework is well established,^20^ leading to highly luminescence nanoparticles that can be tracked by imaging.^21^

Vancomycin, Van, was chosen as it is a well-studied antibiotic, effective against many Gram-positive species but not Gram-negative bacteria which are intrinsically resistant to it due to the low permeability.^2^ This has been attributed to the bulky glycopeptide structure which does not cross the Gram-negative bacteria cell outer membrane. By encapsulation of vancomycin in the silica particle it is envisaged that the interaction with the outer membrane will be governed by the particle properties rather than the properties of the antibiotic. Inclusion of vancomycin in silica nanoparticles has been previously reported through two different methodologies: surface covalent anchoring^22^ or absorption into the porous network of mesoporous silica nanoparticles,^23^ which would not be effective in our case as it would only allow the treatment of those bacterial cells that are in contact with the nanoparticle leading to the aforementioned limitations of usage. In our approach we would use encapsulation of the vancomycin together with the positively charged ruthenium agent during the growth of the silica network.

**Fig. 1:**
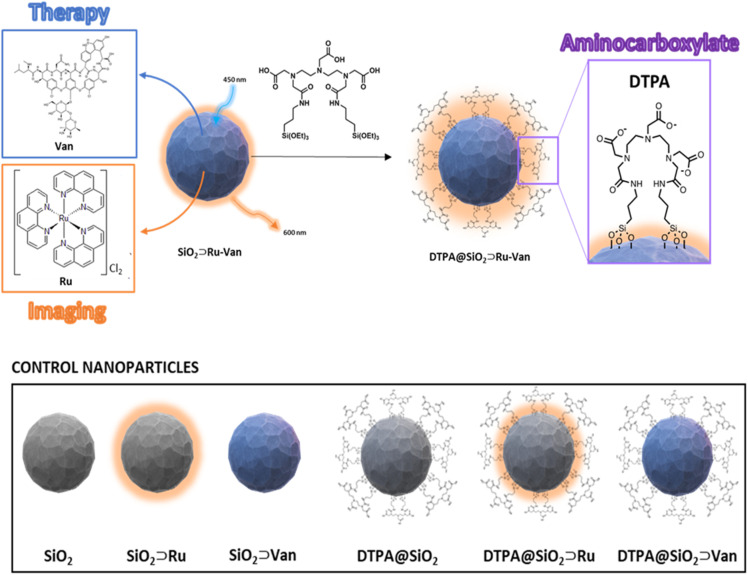
Schematic representation of hybrid DTPA@SiO_2_⊃Ru-Van and nanoparticles with single agents with and without DTPA coating for control experiments.

To modulate the particle's surface properties and interaction with the bacterial membrane we have incorporated a bis(amide) derivative of diethylene triamine pentaacetic acid (noted as DTPA) to attach covalently onto the silica nanoparticle outer surface. Diethylene triamine pentaacetic acid has been shown previously to destabilize the Gram-negative bacterial cell wall, based on its chelating properties to calcium in the bacterial outer membrane.^19–21^ Our hybrid silica design was based on using the surface properties of the particle to induce uptake and the encapsulated antibiotic to act following particle uptake. The hybrid nanoparticle was formed in two steps: firstly, Van was encapsulated together with Ru luminescent agent during the silica nanoparticle formation, followed by the attachment of the surface active DTPA to the isolated nanoparticles to give DTPA@SiO_2_⊃Ru-Van (Fig. 1). To assess the influence of the surface-active ligand and the encapsulation, control nanoparticles were also examined without the surface coating, or with a single agent. All particles were tested against *S. aureus* and a selection of Gram-negative bacterial species and luminescence microscopy was also used to demonstrate tracking of the particles.

## Results and discussion

### Particle formation and characterization

**Fig. 2:**
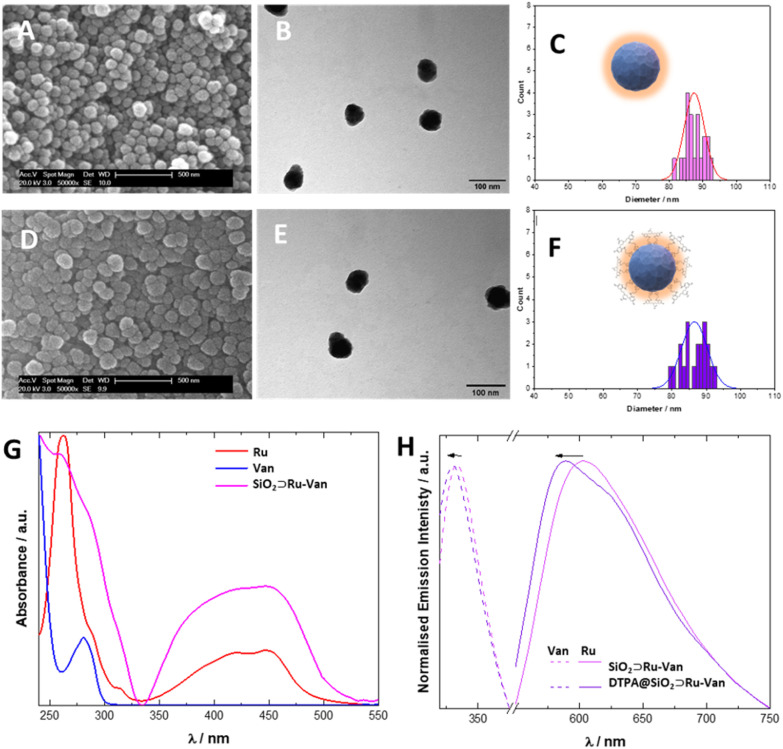
Characterisation and analysis of size and optical properties of hybrid particles with cargo agents Scanning Electron Microscopy (A and D) and Transmission Electron Microscopy (B and E) images of hybrid particles SiO_2_⊃Ru-Van (A and B) and DTPA@SiO_2_⊃Ru-Van (D and E). Size distributions are calculated based on 20 measurements and distribution is fitting according to a Gaussian distribution (C and F). Optical properties of DTPA@SiO_2_⊃Ru-Van and SiO_2_⊃Ru-Van confirming the presence of Van and Ru by solid state UV/vis spectroscopy (G) and solid-state luminescence (H) of Van (*λ*_exc_ = 270 nm) and Ru (*λ*_exc_ = 450 nm).

Our synthetic methodology was based on the formation of the silica nanoparticles in the presence of the antibiotic, Van, and the luminescent tracking agent, Ru under controlled conditions^24^ to yield SiO_2_⊃Ru-Van particles (Fig. 1). Although SiO_2_⊃Ru have been previously reported^20,24^ with the ruthenium complex as encapsulated agent, hybrid particles with antibiotic have not been previously developed. It was envisaged that the addition of vancomycin at the same time of the ruthenium agent will allow growth of silica around them as it occurs with single agents. Encapsulation of both agents may have been driven by the electrostatic properties of the positively charged ruthenium and the hydrogen-bonding of vancomycin with the silica framework. Particles with single agent were also prepared, SiO_2_⊃Ru and SiO_2_⊃Van, under the same conditions for comparisons. Following isolation and purification of the particles with the encapsulated agents, the surface of the particles was modified upon reaction with the silyl surface active bis(amide) derivative (Fig. 1).^25^ The final particles were purified by washing and centrifugation. Size and properties of all nanoparticles were analyzed by a suite of characterization techniques, including Dynamic Light Scattering (DLS) and electron microscopy techniques. A summary of the two hybrid nanoparticle systems is presented in Table 1 and the rest of the nanoparticle systems in Tables S1 and S2.† DLS results provide the hydrodynamic diameter of the particles. In water there is not a significant difference of the coated DTPA@SiO_2_⊃Ru-Van and uncoated SiO_2_⊃Ru-Van particles as it is expected. However, in PBS DTPA@SiO_2_⊃Ru-Van show larger sizes on intensity values without increase of polydispersity which excludes aggregation. The effect is more pronounced with the coated particles which have free carboxylate groups and it is also evidenced upon addition of excess NaCl (Table S1†). The change of the surface coating was also evidenced by the *ζ*-potential measurements with a higher negative potential of DTPA@SiO_2_⊃Ru-Van, −24 mV compared to SiO_2_⊃Ru-Van from −18 mV (Table 1), attributed to the negatively charged carboxylate moieties. This trend in the *ζ*-potential measurements is also supported for the nanoparticles with the single agent Ru or Van upon coating (ESI†). Scanning electron microscopy (SEM) and transmission electron microscopy (TEM) (Fig. 2 and ESI†) images confirmed the uniform, spherical morphology and good monodispersity of SiO_2_⊃Ru-Van and DTPA@SiO_2_⊃Ru-Van (ESI†). Independent particle measurements of the nanoparticles from the microscopy images led to estimated average sizes of 85 ± 3 nm and 87 ± 4 nm for SiO_2_⊃Ru-Van and DTPA@SiO_2_⊃Ru-Van, respectively. The smaller sizes from the microscopy studies were anticipated as the images do not reflect the outer surface of the particle accounted in the hydrodynamic measurements. The same trend for the size and shape of the particles was observed for the particles including the single agents with sizes of 50–90 nm. FT-IR and Raman spectroscopy were employed to provide signatures of the agents in the particles. FT-IR spectra are dominated by the Si–O–Si and Si–O stretches and the individual cargo peaks are too weak to be observed. However, the DTPA stretches are observed in all coated particles with peaks attributed to C–H and N–H bands, as well as characteristic C=O stretches (SI†). The characteristic cargo peaks are observed by Raman spectroscopy. In cargo-loaded SiO_2_ nanoparticles, Raman shifts assigned to the C–C stretching and C–H of both cargos were observed in the 1000–1700 cm^−1^ range (ESI†), confirming their presence in the silica framework.

**Table 1:** Summary of nanoparticle properties for SiO_2_⊃Ru-Van and DTPA@SiO_2_⊃Ru-Van. Dynamic Light Scattering measurements are performed at 25 °C in MiliQ water and phosphate buffer solution (PBS, 0.1 M, pH 7.4), PDI = Polydispersity Index. Size by TEM is determined based on 20 nanoparticle measurements

		Mean size_DLS_/nm				Mean size_TEM_/nm	ζ-Potential/mV
		Intensity	Volume	Number	PDI		
SiO_2_⊃Ru-Van	Water	89 ± 29	86 ± 12	84 ± 22	0.16	85 ± 3	−16 ± 2
	PBS	98 ± 29	82 ± 26	67 ± 18	0.15		−18 ± 6
DTPA@SiO_2_⊂Ru-Van	Water	94 ± 10	92 ± 15	89 ± 14	0.24	87 ± 4	−20 ± 3
	PBS	113 ± 28	105 ± 25	94 ± 20	0.21		−24 ± 5

To evaluate their properties for detection in bacterial cells, optical spectroscopy was employed. Solid-state UV/vis spectroscopy of SiO_2_⊃Ru-Van showed a broad band centred at 425 nm, characteristic of the singlet metal to ligand charge transfer transition (^1^MLCT) of the Ru complex along with a broad band with shoulder at 281 nm and a 261 nm attributed to the Van antibiotic π–π* transitions and phenanthroline transitions, respectively (Fig. 2). Excitation of both coated and uncoated particles, DTPA@SiO_2_⊂Ru-Van and SiO_2_⊃Ru-Van, at the ^1^MLCT band at 450 nm showed a broad luminescence peak in the red of the spectrum which is characteristic of the ^3^MLCT transition. The peak is observed at 613 and 612 nm in SiO_2_⊃Ru-Van and SiO_2_⊃Ru showing no effect from the presence of Van with a small lifetime increase (ESI†). However, the coated particles DTPA@SiO_2_⊂Ru-Van showed a blue shift to 595 nm. This was further supported by the 19 nm shift of the emission in DTPA@SiO_2_⊃Ru in comparison with SiO_2_⊃Ru. Interestingly, the luminescence lifetime of the Ru-based emission was increased from 1.69 μs in SiO_2_⊃Ru-Van to 2.52 μs in DTPA@SiO_2_⊂Ru-Van and from 1.45 μs in SiO_2_⊃Ru to 3.31 μs DTPA@SiO_2_⊃Ru. The increase of the lifetime may be attributed to the surface functionalisation with DTPA which decreases oxygen access, responsible for quenching the ruthenium luminescence. Upon excitation at 270 nm, a fluorescence signal at 330 nm indicated the presence of Van in SiO_2_⊃Ru-Van, a 3 nm blue sift was observed for Van's emission after coating with DTPA. SiO_2_⊃Van also displayed a 5 nm blue shift upon DTPA coating (ESI†). The hybrid particles display dual emission in the blue from vancomycin and the red from the ruthenium agent with the latter making them attractive for detection in bacterial cells. To quantify the surface coating, we used the DTPA ligand's property to bind to lanthanide ions to form 1 : 1 stable complexes.^26^ By titration of a solution of Tb^3+^ to a solution of DTPA@SiO_2_⊃Ru-Van the luminescence signal of terbium was increased, reaching a plateau. The coordination of terbium to he DTPA moiety on DTPA@SiO_2_⊃Ru-Van, forming a 1 : 1 complex with DTPA, led to replacement of water molecules in the terbium coordination sphere which quench the terbium signal, hence leading to a luminescence signal increase. From the titration, the concentration of DTPA was correlated to the nanoparticle concentration and it was estimated to be 0.53 mg_DTPA_/mg_NP_.

### Loading of the agents in nanoparticles and antibiotic release

**Fig. 3:**
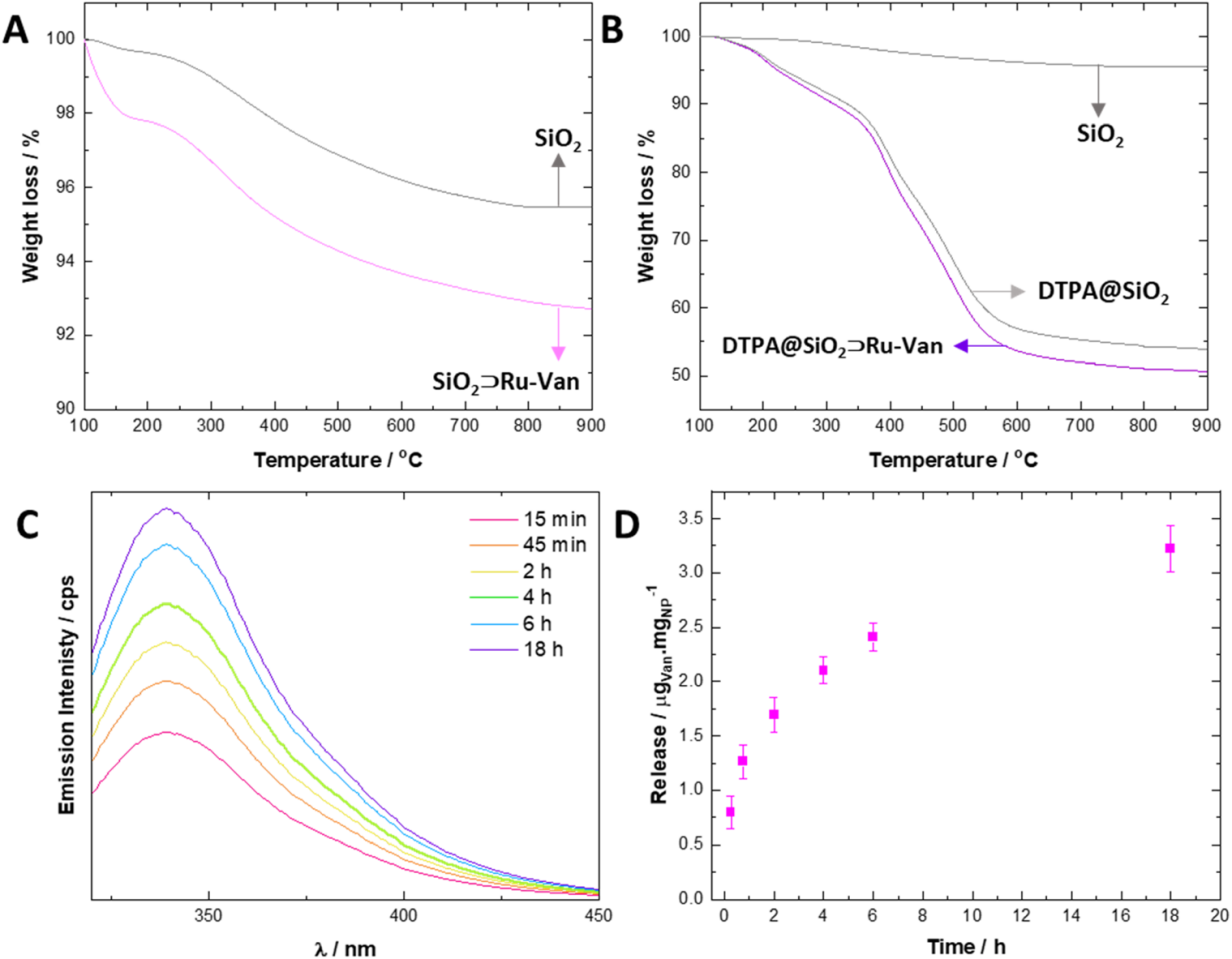
Quantification of included cargo by Thermal Gravimetric Analysis and optical spectroscopy studies showing cargo loading. TGA profiles showing Ru and Van loading (A) and DTPA coating (B) in SiO_2_⊃Ru-Van and DTPA@SiO_2_⊃Ru-Van, respectively. Samples are heated from 30 °C to 900 °C at 1 °C per min rate with a 10 min isothermal step at 100 °C to ensure water removal. All degradation curves are set at 100% for clarity. Fluorescence monitoring of release of Van from hybrid DTPA@SiO_2_⊃Ru-Van nanoparticles and cumulative release graph (C and D) at 37 °C, 150 rpm, in water (*λ*_exc_ = 270 nm and *λ*_em_ = 330 nm) (*n* = 3).

Loading of the agents in SiO_2_⊃Ru-Van were determined by (a) thermogravimetric analysis (TGA) and (b) optical spectroscopy of the remaining supernatant following centrifugation (Fig. 3). TGA is a widely used technique for material characterization and determination of the loading of drugs in silica nanoparticles, based on the continuous monitoring of sample's weight loss upon heating at a defined rate under a controlled atmosphere. In the plain SiO_2_ particles the first loss of weight of 15 μg/mg_NP_ is observed at 150 °C and it is attributed to adsorbed water as previously observed^21^ where the hybrid SiO_2_⊃Ru-Van particles showed a loss of weight from 200–900 °C of 58 μg/mg_NP_ (Fig. 3A); in comparison, the SiO_2_⊃Ru particles showed a weight loss of 0.3% and SiO_2_⊃Van a loss of 8.9%, although the latter did not show distinct steps as the other particles, attributed to Van thermal degradation.

Interestingly, the TGA graphs of the particles coated with DTPA, DTPA@SiO2⊃Ru-Van, DTPA@SiO2⊃Ru and DTPA@SiO_2_⊃Van were dominated by the degradation of the DTPA with an estimated content of 0.55 mg_DTPA_/mg_NP_ (Fig. 3B) in agreement with the independent optical analysis by coordination of Tb^3+^ which was 0.53 mg_DTPA_/mg_NP_. For the control nanoparticles, the DTPA content by TGA was calculated also to be between 0.49 to 0.55 mg_DTPA_/mg_NP_ (ESI†). In order to define the amount of Ru and Van separately in SiO_2_⊃Ru-Van we studied the luminescence signal of the supernatant Ru and Van agents, following centrifugation after their preparation. It was estimated that in SiO_2_⊃Ru-Van there were 5 μg_Ru_/mg_NP_ and 54 μg_Van_/mg_NP_.

These were in good agreement with the overall agent content determined by TGA (0.6 μg_Agent_/mg_NP_).

Release of the antibiotic Van from the hybrid nanoparticles was monitored by fluorescence spectroscopy when the particle dispersion was subjected to shaking (150 rpm) at 37 °C. It was calculated that up to 3.3 ± 0.5 μg_Van_/mg_NP_ of the antibiotic can be released from DTPA@SiO_2_⊃Ru-Van with a continuous cumulative release (Fig. 3C and D), up to 18 h, determined based on calibration of Van's fluorescence signal upon excitation at 270 nm. DTPA@SiO_2_⊃Van showed a cumulative release of 3.8 ± 0.3 μg_Van_/mg_NP_ upon continuous shaking. Minimal release of the antibiotic Van was observed under static conditions (ESI†). Furthermore, the luminescent agent's release Ru under shaking or static conditions led also to minimal leakage.

### Antibacterial activity

In order to assess the antibacterial properties of the designed luminescent hybrid nanoparticles in the first instance, *S. aureus* (ATTC23841) and *E. coli* (MG1665) were chosen as representative Gram-positive and Gram-negative bacterial species, respectively. Gram-negative bacteria have a double membrane structure that includes a rigid, impermeable outer membrane making them intrinsically resistant to many antibiotics including vancomycin. Gram-positive bacteria, however, possess a much simpler single cell membrane through which many more antibiotics can pass.^1,2,28^

The antibacterial activity of all the nanoparticles and individual agents was evaluated by measuring the Minimum Inhibitory Concentration (MIC) in Lysogeny Broth (LB) with mild shaking. MIC values were determined as the minimum nanoparticle concentration (mg mL^−1^) where no bacterial growth was observed (Table 2).

**Table 2:** Minimal Inhibitory Concentrations (MIC, mg mL^−1^) of Van, SiO_2_, DTPA@SiO_2_, SiO_2_⊃Ru-Van and DTPA@SiO_2_⊃Ru-Van for *S. aureus* (ATTC23851), *E. coli* (MG1665), *A. baumannii* (AC05), *K. pneumoniae* (ATCC43816R), *K. oxytoca* (080-KPC CHD), *C. feundii* (NCTC9750) and *S. enterica* (SL1344) determined by the 2-fold dilution method upon shaking at 150 rpm at 37 °C in LB media. Depicted results are based on three biological replicas

	*E. coli*	*A. baumannii*	*K. pneumoniae*		*K. oxytoca*	*C. freundii*	*S. enterica*	*S. aureus*
Chelating particles DTPA@SiO_2_⊃Ru-Van	1.25 (4.1)^a^	1.25 (4.1)^a^	0.75 (2.5)^a^		1.25 (4.1)^a^	1.25 (4.1)^a^	0.75 (2.5)^a^	0.37 (1.2)^a^
Plain antibiotic Van	> 0.25	> 0.25	> 0.25		> 0.25	> 0.25	> 0.25	0.001
Non-chelating particles SiO_2_⊃Ru-Van	5.00	2.50	2.50		5.00	5.00	2.50	1.25

a Estimated Van released based on release studies of DTPA@SiO_2_⊃Ru-Van*in vitro* (in μg mL^−1^, ± 16%).

Chelating nanoparticles with the cargos, DTPA@SiO_2_⊃Ru-Van, displayed significantly increased antimicrobial activity (decreased MIC values) in both *S. aureus* (MIC = 0.37 mg mL^−1^) and *E. coli* (1.25 mg mL^−1^) compared to SiO_2_⊃Ru-Van suggesting that these functionalised nanoparticles had increased uptake into *E. coli*. It is worth noting that the estimated concentration of the released antibiotic is only by DTPA@SiO_2_⊃Ru-Van, is in the μg ml^−1^ region (Table 2), showing the efficiency of the delivery system. As expected, the antibiotic Van only had antimicrobial activity against *S. aureus*, (MIC = 1 μg mL^−1^), but not *E. coli* reflecting the intrinsic low permeability of the Gram-negative outer membrane. Similarly, the luminescent tracking agent, Ru, had moderate antimicrobial activity against *S. aureus* (MIC = 312 μg mL^−1^, Table S5†) but not *E. coli* or the other Gram-negative species. The plain SiO_2_ nanoparticles were also not found with antibacterial activity up to a concentration of 10 mg mL^−1^ (Table S5†). Inclusion of the cargos in the nanoparticles to give SiO_2_⊃Ru-Van, SiO_2_⊃Ru or SiO_2_⊃Van, show only moderately increased bacterial susceptibility, suggesting that encapsulating within the nanoparticle had not sufficiently increased penetration of the antimicrobial agent. The particles with no cargo, DTPA@SiO_2_, had no antimicrobial activity against *E. coli*. Antimicrobial activity of antibiotic containing nanoparticles was greater when particles were shaken compared with static incubation which was in good agreement with the release studies under static conditions (ESI†). A range of other clinically relevant Gram-negative bacterial species were also analysed (Table 2) to examine the effect of the aminocarboxylate coating. The results show that functionalisation of nanoparticle surface greatly increased susceptibility in all Gram-negative species tested: *A. baumannii* (AC05), *K. pneumoniae* (ATCC43816R), *K. oxytoca* (080-KPC CHD), *C. feundii* (NCTC9750) and *S. enterica* (SL1344). In these Gram-negative bacterial species, the antibiotic had no effect if delivered without the nanoparticle system even when delivered at concentrations of 0.25 mg mL^−1^. Overall DTPA@SiO_2_⊃Ru-Van shows greater antibacterial activity than previously achieved with vancomycin encapsulated in porous networks.^23^ Moreover, all functionalised nanoparticles showed low overall cytotoxicity against A549 lung cancer cells as assessed by the MTT assay. Cell viability was above 85% at concentrations used in MIC experiments and this was not statistically significant as assessed by a 1-way ANOVA followed by a post-hoc Tukey *T*-test (Fig. S12†).

We attribute the activity of the hybrid nanoparticles DTPA@SiO_2_⊃Ru-Van to the DTPA chelating ability against Ca^2+^ and Mg^2+^. It has been previously reported that polyaminocarboxylate chelators interact with bacterial membranes and damage its structural stability by removing crucial Ca^2+^ and Mg^2+^ cations.^27,28^ Recent simulation studies by L. A. Clifton *et al.*^29^ on the effect of ethylene diamino tetraacetic acid in Gram-negative bacteria cell membrane revealed that cation removal leads to an increasing presence of phospholipids in the outer membrane structure, along with a decreasing concentration of lipopolysaccharides. Furthermore, the binding of cations to bisamides of diethylene triamino pentaacetic acid is known to be in the range of 10^5^–10^9^.^30^ Isothermal titration calorimetry (ITC) experiments allowed the evaluation of the binding of DTPA@SiO_2_⊃Ru-Van particles to Ca^2+^ which showed a log *K* value of 7.5 ± 0.6 (ESI†), in good agreement with reported values. The chelating effect of DTPA@SiO_2_⊃Ru-Van was further confirmed by MIC studies of the particles DTPA@SiO_2_⊃Ru-Van in the presence of Ca^2+^. The chelated particles (Ca^2+^DTPA@SiO_2_⊃Ru-Van) showed a lower efficacy of the nanoparticles with values similar to the uncoated SiO_2_⊃Ru-Van (Table S7†). These results confirmed the blocking the chelating ability of the nanoparticles, did not lead to efficient antibiotic delivery.

### Imaging in bacterial cells

**Fig. 4:**
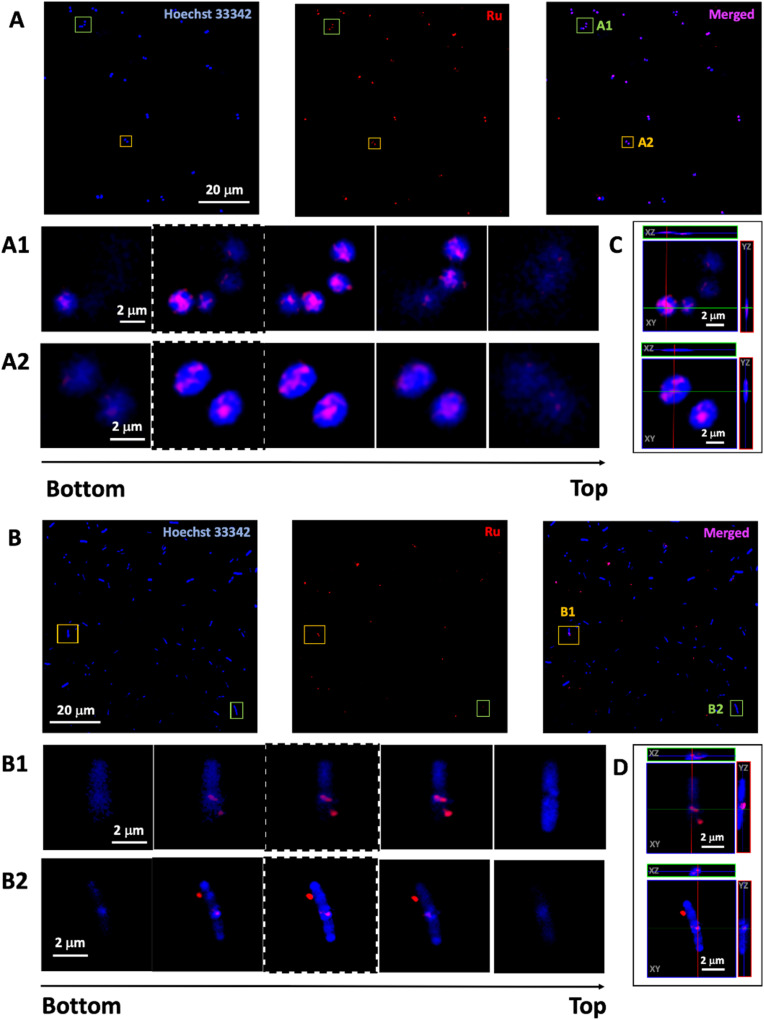
Live bacteria cell imaging (*n* = 5) by structural illumination microscopy showing red luminescent particle uptake in *S. aureus* (A) and *E. coli* (B). Overnight grown bacteria are treated with DTPA@SiO_2_⊃Ru-Van (2.5 mg mL^−1^) for 2 h at 37 °C under mild shaking (150 rpm). Images show each channel for the outline of bacteria cells (blue) and particle luminescence (red) as well as merged channels. 3-D reconstruction: image sequence extracted from the z-stack of two cells in *S. aureus* (A1 and A2) and in *E. coli* (B1 and B2) and orthogonal reconstruction *S. aureus* (C) and *E. coli* (D) of central plane image (highlighted with dashed line) showing red luminescence from DTPA@SiO_2_⊃Ru-Van inside the cells. Red channel, ruthenium emission (*λ*_exc_ = 488 nm, *λ*_em_ = 580–620 nm); blue channel, Hoechst H33342 emission (*λ*_exc_ = 405 nm, *λ*_em_ = 410–455 nm).

In order to confirm that increased antimicrobial activity of the particles was due to increased penetration, luminescence microscopy was used to monitor the uptake of the emissive particles into bacterial cells based on the detection of the ruthenium signal. The DTPA@SiO_2_⊃Ru-Van and SiO_2_⊃Ru-Van were incubated with *S. aureus* or *E. coli* and images were collected of the live bacteria cells (Fig. 4). Structural illumination microscopy images showed the uptake of the red luminescent particles by tracking the signal of the Ru encapsulated cargo in spherical, *S. aureus*, and rod-shaped, *E. coli*., bacterial cells co-stained with nucleic acid dye, Hoechst H33342. Investigation of the penetration of the particles into the bacteria was studied by z-stack imaging. An intensity profile graph of the ruthenium signal in comparison with Hoechst H33342 as a bacterial cell stain indicated particle presence inside the bacterial cells. *S. aureus* bacteria cells show particle uptake based on the Ru luminescence signal for both DTPA uncoated- and coated-particles, namely SiO_2_⊃Ru-Van (ESI†) and DTPA@SiO_2_⊃Ru-Van (Fig. 4). This is expected due to their simpler cell membrane which allows particle penetration. This result is also in agreement with the MIC assays which show activity of both set of particles.

However, *E. coli* bacterial cells, show that only the DTPA coated particles, DTPA@SiO_2_⊃Ru-Van, were visualised inside the stained cells, by monitoring of the ruthenium signal (Fig. 4). Penetration was confirmed by z-stack 3D image reconstruction of selected cells and orthogonal views (Fig. 4 and S8 and S9†). Absence of DTPA surface functionalization resulted in no particle penetration in *E. coli* (Fig. S10†). Quantification of the proportion of bacterial cells with internalised DTPA@SiO_2_⊃Ru-Van was performed by determining the colocalization of the red luminescent particles and blue-stained bacterial cells using ImageJ software based on previous methods.^31^ This analysis showed that un-coated/non-functionalised nanoparticles (SiO_2_⊃Ru-Van) did not enter any *E. coli* cells while DTPA@SiO_2_⊃Ru-Van were found in 45% of *E. coli.* Analysis of the same live cell imaging of *E. coli* treated with DTPA@SiO_2_⊃Ru-Van for 18 h (Fig. S11†) show that nanoparticle penetration is increased to 70 ± 7% of the bacteria cells. These conclusions are further supported by the JACoP analysis (Table S6†) showing accuracy of the colocalization analysis between both channels, indicating presence of the particles within the bacteria cells which is increased after longer incubation time. This supports the results of the higher antibacterial activity of DTPA@SiO_2_⊃Ru-Van which are used as vehicles to transport Van across the Gram-negative bacterial membrane and release the antibiotic inside the bacterial cells.

## Conclusions

The dual encapsulation of a glycopeptide antibiotic and a ruthenium complex was successful, allowing nanoparticles to be tracked in the bacterial cells by the ruthenium-based red signal. This nanoparticle platform was ideal for the modification of the outer surface with a membrane permeating agent, such as DTPA, which allowed the DTPA@SiO_2_⊃Ru-Van nanoparticles to be internalized in the Gram-negative bacterial species and release a therapeutic agent, which was otherwise not able to permeate Gram-negative bacterial cells. The permeability of the nanoparticles is attributed to the chelating properties of the aminocarboxylate surface group, DTPA, which is known to bind to calcium and magnesium ions. These results suggest that encapsulation of antibiotics into a nanoparticle coated with chelating ligands shows strong potential for increasing the spectrum of activity of existing or novel drugs that are not able to permeate Gram-negative bacterial cells. Indeed, DTPA@SiO_2_⊃Ru-Van shows greater antibacterial activity than any previously reported platforms for vancomycin delivery. Our results represent a step forward against Gram-negative bacterial infections to allow nanoparticle designs with imaging and therapeutic agents to be internalized in cells, overcoming membrane impermeability challenges and tracking drug delivery in cells.

## Author contributions

A. R. M. performed all experimental work with A.di M. contributing in imaging analysis and NJH in MTT assays, J. M. A. B. and Z. P. originally designed the project with A. R. M. providing input; all authors contributed to versions of the manuscript.

## Conflicts of interest

The authors declare no conflict of interest.

## Supplementary Material

NA-005-D2NA00884J-s001
