# Supplementary material for: Chelating silica nanoparticles for efficient antibiotic delivery and particle imaging in Gram-negative bacteria
Source: Nanoscale Adv. 2023 Feb 20;5(9):2453–61. doi: 10.1039/d2na00884j (PMC10153079; doi:10.1039/d2na00884j)
Supplement: NA-005-D2NA00884J-s001 [file NA-005-D2NA00884J-s001.pdf]

Electronic Supplementary Material (ESI) for Nanoscale Advances.
This journal is © The Royal Society of Chemistry 2023

Supplementary Information

Chelating silica nanoparticles for efficient antibiotic delivery and particle imaging in Gram-negative bacteria

Asier R. Muguruza, Alessandro di Maio, Nikolas J. Hodges, Jessica M.A. Blair and Zoe Pikramenou

Prof. Zoe Pikramenou
Email:z.pikramenou@bham.ac.uk

Dr J. M. A. Blair
Email: J.M.A.Blair@bham.ac.uk

This PDF file includes:

Materials and Methods
Figures S1 to S15 and Tables S1 to S7
with

- Further characterisation data of all silica nanoparticles and DTPA agent used in control experiments (size, optical properties);
- MIC results (of control agents and nanoparticles);
- luminescent properties of the included agent in different particle environments;
- Quantification of the DTPA and cargos, Ru and Van, in control particles;
- MTT assays of all nanoparticles;
- Further structural illumination images of bacteria loaded with nanoparticles.
- ITC experiments

Materials and Methods

Materials and Instrumentation. $Ru(phen)_3Cl_2·6H_2O$ (Ru) and tetraethoxysilane (TEOS) were purchase from Sigma-Aldrich and Vancomycin·HCl (Van) from Alfa Aesar. Rest of chemicals and consumables were purchased from Sigma-Aldrich and were of the highest quality available. ^{1}H-NMR spectra were recorded on a Bunker AV300 (300 MHz) spectrometer using d_6-DMSO as solvent. ESI-MS was recorded using a Thermo Scientific LCQ Fleet ion trap LC/MS mass spectrometer equipped with a Finningham Surveyor autosampler and controlled by XCalibur software. FT-IR data was recorded with a Perkin Elmer 100FT-IR spectrometer fitted with ATR attachment; samples were dried overnight under vacuum prior the measurements. DLS data was recorded by a Malvern Panalytical Zetasizer ZS instrument equipped with a H-Ne 633 nm laser at 25 ºC and with a back-scattering of 173 º using PBS (0.1 M, pH = 7.4) as dispersant. The instrument was controlled with a Malvern DTS 7.03 software. All sizes were determined in based on 5 measurements containing 10 runs on each. ζ–potential was determined after measuring each sample at 25 °C in triplicate. Solid-state UV-Vis spectra were recorded using a Cary 5000 Uv-Vis spectrophotometer equipped with a solid-state attachment. Steady state luminescence measurements and time resolved studies were recorded on an Edinburgh Instruments FLS920 steady state and time-resolved spectrometer. The detection system used an incorporated R928 Hamamatsu photomultiplier tube. F980 spectrometer analysis software was used to record the data, and all spectra were corrected for photomultiplier and instrument response. Luminescent lifetimes were recorded with EPL-375 laser as an excitation source and fitted using Edinburgh Instruments FAST software with estimated error of ± 10%. Determination of released cargo was determined using a Horiba TCSPC Fluorolog spectrophotometer with Fluor Essence V3.8 software, corrected for lamp and instrument response. Thermogravimetric analysis was performed in a TGA 8000 PerkinElmer thermogravimetric analyser, controlled by a Pyris Software version 13.3.1.0019. Samples were loaded into ceramic sample crucibles, circa 6.6 mm in diameter and 1.95 mm in height. The temperature is held at 30 ºC for 1 min then increased from 30 to 100 ºC at 100 ºC min^{-1}, followed by an isothermal step at 100 ºC for 5min. Then, temperature was raised from 100 to 380 ºC at 5ºC min^{-1}, holding at 380 ºC for 10 min followed by a 5ºC min^{-1} rate from 380ºC to 800 ºC. Finally, samples were cooled down from 800 ºC to 30 ºC at a 30 ºC min^{-1} rate. All thermal decompositions are performed under oxygen flow. Isothermal titration calorimetry (ITC) was performed using a Malvern MicroCal PEAQ-ITC instrument and the binding curve was fitted with the Malvern MicroCal PEAQ-ITC V1.41 using the 'One Set of Sites' model. Structural illumination imaging was performed using a Zeiss Elyra 7 system equipped with 405 nm and 488 nm lattice lasers. Structural illumination images were processed using the Zeiss ZEN software and analysed using the ImageJ V1.51 software. Colocalization analysis between channels was carried out using the Fiji image software plugin JACoP (3), from which the Pearson and Manders' Overlapping correlation parameters were calculated over five different images per treatment.

Synthesis of $SiO_2 \supset$Ru-Van. $Ru(phen)_3Cl_2$ (Ru) and Vancomycin HCl (Van) loaded silica nanoparticles (SiO_2) were synthetized following a modified methodology reported by D. J. Lewis *et al.*(1) Briefly, a solution containing 25 mL of EtOH, 1.5 mL of 28% NH_4OH and 125 μL of a 13 mM aqueous solution of Ru and 125 μL of a 67 mM aqueous solution of Van was stirred at room temperature. To this, a solution containing 5 mL of EtOH and 2 mL of TEOS was slowly added, and the resulting solution was stirred for 2 h at room temperature, followed by 15 min of sonication treatment (200 W, 50 Hz) before another hour of stirring. Finally, obtained yellow suspension was centrifuged (7450 rpm, 15 min) and the supernatant removed. **$SiO_2 \supset$Ru-Van** were washed with water (3 x 20 mL) and dried overnight under vacuum.

Synthesis of $SiO_2 \supset$Van. Van loaded SiO_2 were synthesised following the same procedure by using 125 μL of a 67 mM aqueous solution of **Van**.

Synthesis of $SiO_2 \supset$Ru. Ru loaded SiO_2 were synthesised following the same procedure by using 125 μL of a 13 mM aqueous solution of **Ru**.

Synthesis of silyl-derivative bisamide diethyelenetriaminepentaacetic acid, noted as DTPA. Diethylenetriaminepentaacetic acid dianhydride (0.5 g, 1.4 mmol) was dissolved in anhydrous pyridine (20 mL) under nitrogen. To the stirred solution, 3-aminopropyl triethoxysilane (APTES, 0.7 mL, 2.8 mmol) was added. The solution was left to stir under nitrogen for 24 h at room temperature. The resulting solution was reduced to 5 mL upon rotary evaporation. The product was precipitated using anhydrous hexane (200 mL), stirred in an ice bath under nitrogen. The product was left to further precipitate overnight at 4 °C. Resulting white precipitate was isolated via filtration, washed with anhydrous hexane (4 x 40 mL) and left to air dry (1 g, 95%). ^{1}HNMR (300 Hz, d_6-DMSO) 8.08 (2H, s, NHCO), 3.73 (12H, q, J = 6.96, OCH_2CH_3), 3.33 (6H, s, CH_2COOH), 3.22 (4H, s, NH_2CON), 3.04 (4H, m, J = 7.11, NCH_2Et), 2.94 (4H, t, J = 6.16, NCH_2CH_2N), 2.81 (4H, t, J = 6.16, NCH_2CH_2N), 1.45 (4H, m, J = 7.11, $NCH_2CH_2CH_2$), 1.14 (18H, t, J = 6.96, CH_3), 0.52 (4H, t, J = 7.5, $NCH_2CH_2CH_2$). ESI-MS: m/z 800.400 ($[M-H]^-$, calcd. 800.022). FTIR (cm^{-1}) = 3215 (w, N-H), 2965 (m, C-H_2), 2920 (m, C-H_2), 2875 (m, N-H), 1653 (s, C=O), 1355 (m, C-OCH_2CH_3), and 1073 (s, C-O).

Synthesis of DTPA@SiO_2. The **SiO_2** nanoparticles (30 mg) and silyl-derivative bisamide DTPA (70 mg) were mixed in anhydrous toluene (10 mL) and sonicated for 5 min to give a homogenous suspension which was heated in reflux at 125 °C under nitrogen overnight. The **DTPA@SiO_2** nanoparticles were isolated by centrifugation and washed with ethanol (1 x 20 mL) and MiliQ water (3 x 20 mL) to remove unreacted DTPA-Si_2. FTIR (cm^{-1}) = 3215 (w, N-H), 2965 (m, C-H_2), 2920 (m, C-H_2), 2875 (m, N-H), 1653 (s, C=O), and 1073 (s, C-O).

Quantification of DTPA on SiO_2 by Tb(III) titration on DTPA@SiO_2. In order to quantify surface conjugated DTPA molecules, a methanolic solution of $TbCl_3{\cdot}6H_2O$ (0.33 mM) was added in 5 ml aliquots to a suspension of **DTPA@SiO_2** (1 mg in 2 mL of MeOH) and the Tb(III) luminescence signal (λ_{exc} = 236 nm, λ_{em} = 450 – 750 nm) was monitored.

Release studies. Release studies were performed using loaded SiO_2 suspension (2.5 mg/mL) in Milli-Q water at 37 °C under mild shaking (150 rpm) at 37 °C. Aliquots were removed at different time points, centrifuged (5 min at 14000 rpm) to remove nanoparticles and released Ru and/or Van in the supernatant determined by fluorescence spectroscopy using a Horiba Fluorolog-3 spectrometer (for Ru λ_{exc} = 450 nm and λ_{em} = 600 nm and Van λ_{exc} = 270 nm and λ_{em} = 330 nm).

Minimal Inhibitory Concentration (MIC) determination. MIC values of studied samples, Ru and Van were determined for *S. aureus* (ATCC29213) *E. coli* (MG1655), *A. baumannii* (AC05), *K. pneumoniae* (ATCC43816R), *K. oxytoca* (080-KPC CHD), *C. feundii* (NCTC9750) and *S. enterica* (SL1344) via the standard broth-dilution method in 96-well microlitre plates in Lysogeny Broth (LB). The MIC of each compound was evaluated using a 2-fold decreasing concentration of each sample between 500 – 0.5 μg/mL for Ru and Van and between 10 – 0.04 mg/mL for the **SiO_2** nanoparticles against a bacterial inoculum of 10^7 - 10^9 colony forming units per mL (CFU/mL). Plates were incubated at 37 °C for 18 h under mild shaking (150 rpm). MIC values were determined as the lowest concentration where no turbidity is observed.

Isothermal Titration Calorimetry (ITC). Binding constant of Ca^{2+} with **DTPA@$SiO_2 \supset$Ru-Van** was determined by ITC in PBS (0.1 M, pH = 7.4) at 25 °C titrating a solution of $CaCl_2$ (2.5 mM) into a **DTPA@$SiO_2 \supset$Ru-Van**

suspension with a DTPA concentration of 0.32 mM. Recorded data was fitted using the Malvern MicroCal PEAQ-ITC V1.41 software.

Structural illumination microscopy (SIM) imaging. In order to study the cell-uptake of nanoparticles, overnight inocula (2 mL) were mixed with **SiO_2⊃Ru-Van** and **DTPA@ SiO_2⊃Ru-Van** with a final nanoparticle concentration of 2.5 mg/mL and incubated at 37 ºC and 150 rpm for 2 h or 18 h. 1 mL of incubated cells were stained with Hoechst H33342 (10 μL, 1 mM) for 30 min, followed with washing with PBS (0.1 M, pH 7.4, 1 mL). Stained bacteria were placed on microscope slides and imaged. In each experiment five images were obtained from five independent biological replicates (n=5).

MTT (3-(4,5-dimethylthiazol-2-yl)-2,5-diphenyltetrazolium bromide) cell viability assays. Cytotoxicity of studied nanoparticles was assessed by MTT cell viability assays using A459 lung cancer cell line. Cells were seeded at 6500 cells per plate in 96-well plates and left overnight for attachment to occur. The next day the media was removed and fresh media (100 μL) containing nanoparticles added. Following treatment for 18 h the media was removed, and cells incubated with 200 μL of media containing 0.5 mg mL^{-1} MTT solution for 3 hours. The media was removed and reduced MTT was solubilized by addition of DMSO (200 μL) and absorbance at 590 nm measured in a plate reader (Tecan Infinite 200Pro) as an indicator of mitochondrial reductive activity and cellular viability. All samples were normalized to a solvent (DMSO) blank and the results represent the mean of three experiments carried out in triplicate (n=3±SEM).

Table S1. Dynamic Light Scattering (DLS) and surface ζ–potential measurements of **SiO_2**, **SiO_2⊃Ru**, **SiO_2⊃Van** and respective nanoparticles. Measurements were performed at 25 ºC in PBS buffer (0.1 M, pH = 7.4, $[Na^+]$ = 153 mM) with SiO_2 concentration of 1 mg/mL. Additional data was recorded in water and in PBS with addition of NaCl and tetrabutylammonium chloride (Bu_4NCl) to explore the effect of the ionic strength of the media in the hydrodynamic size of the hybrid nanoparticles.

	Dispersant	Size / nm				ζ-potential / mV
		Intensity	Volume	Number	PDI	
**SiO_2**		68 ± 8	65 ± 5	57 ± 2	0.02	-37 ± 6
**DTPA@SiO_2**		89 ± 33	67 ± 26	52 ± 15	0.17	-45 ± 7
**SiO_2⊃Ru**		74 ± 20	61 ± 18	51 ± 12	0.23	-25 ± 5
**DTPA@SiO_2⊃Ru**		121 ± 26	113 ± 28	100 ± 22	0.24	-37 ± 6
**SiO_2⊃Van**		94 ± 22	64 ± 18	47 ± 17	0.07	-24 ± 9
**DTPA@SiO_2⊃Van**		103 ± 30	92 ± 30	81 ± 15	0.17	-40 ± 7
**SiO_2⊃Ru-Van**	**Water**	89 ± 29	86 ± 12	84 ± 22	0.16	-16 ± 2
	**Water + 153 mM NaCl**	100 ± 21	89 ± 15	72 ± 23	0.17	
	**PBS**	98 ± 29	82 ± 26	67 ± 18	0.15	-18 ± 6
	**PBS + 500 mM NaCl**	168 ± 22	148 ± 21	115 ± 15	0.18	
	**PBS + 500 mM Bu_4NCl**	148 ± 22	139 ± 15	148 ± 20	0.20	
**DTPA@SiO_2⊃Ru-Van**	**Water**	94 ± 10	92 ± 15	89 ± 14	0.24	-20 ± 3
	**Water + 153 mM NaCl**	118 ± 15	110 ± 21	98 ± 23	0.19	
	**PBS**	113 ± 28	105 ± 25	94 ± 20	0.21	-24 ± 5
	**PBS + 500 mM NaCl**	185 ± 14	175 ± 31	170 ± 30	0.32	
	**PBS + 500 mM Bu_4NCl**	165 ± 17	155 ± 23	151 ± 22	0.25	

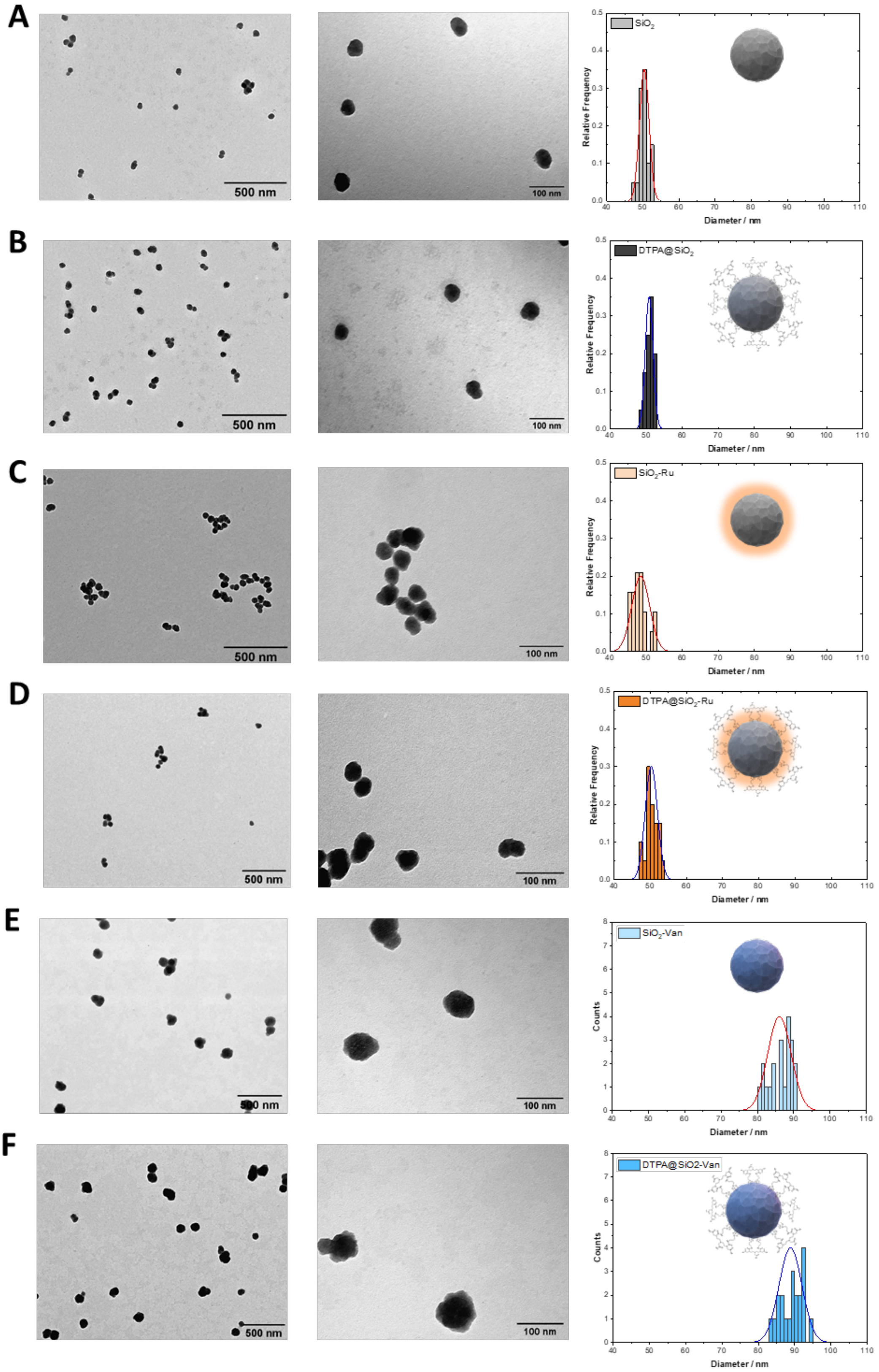

Figure S1. Transmission Electron Microscopy images and particle size distribution of (A) **SiO_2**, (B) **DTPA@SiO_2**, (C) **$SiO_2 \supset$Ru**, (D) **DTPA@$SiO_2 \supset$Ru**, (E) **$SiO_2 \supset$Van** and (F) **DTPA@$SiO_2 \supset$Van**. Size-distribution was calculated using a Gaussian distribution function based on 20 particle measurements.

Table S2. Particle sizes according to TEM images. Average value and standard deviation are calculated using 20 particles. Polydispersity index (PDI) is determined as (standard deviation)^2/mean value.

Table S2. Particle sizes according to TEM images. Average value and standard deviation are calculated using 20 particles. Polydispersity index (PDI) is determined as (standard deviation)^2/mean value.

Sample	$Size_{TEM}$ / nm	PDI
**SiO_2**	50 ± 1	0.02
**DTPA@SiO_2**	51 ± 1	0.02
**SiO_2⊃Ru**	48 ± 3	0.03
**DTPA@SiO_2⊃Ru**	50 ± 2	0.03
**SiO_2⊃Van**	84 ± 5	0.02
**DTPA@SiO_2⊃Van**	88 ± 6	0.02
**SiO_2⊃Ru-Van**	85 ± 3	0.02
**DTPA@SiO_2⊃Ru-Van**	87 ± 4	0.02

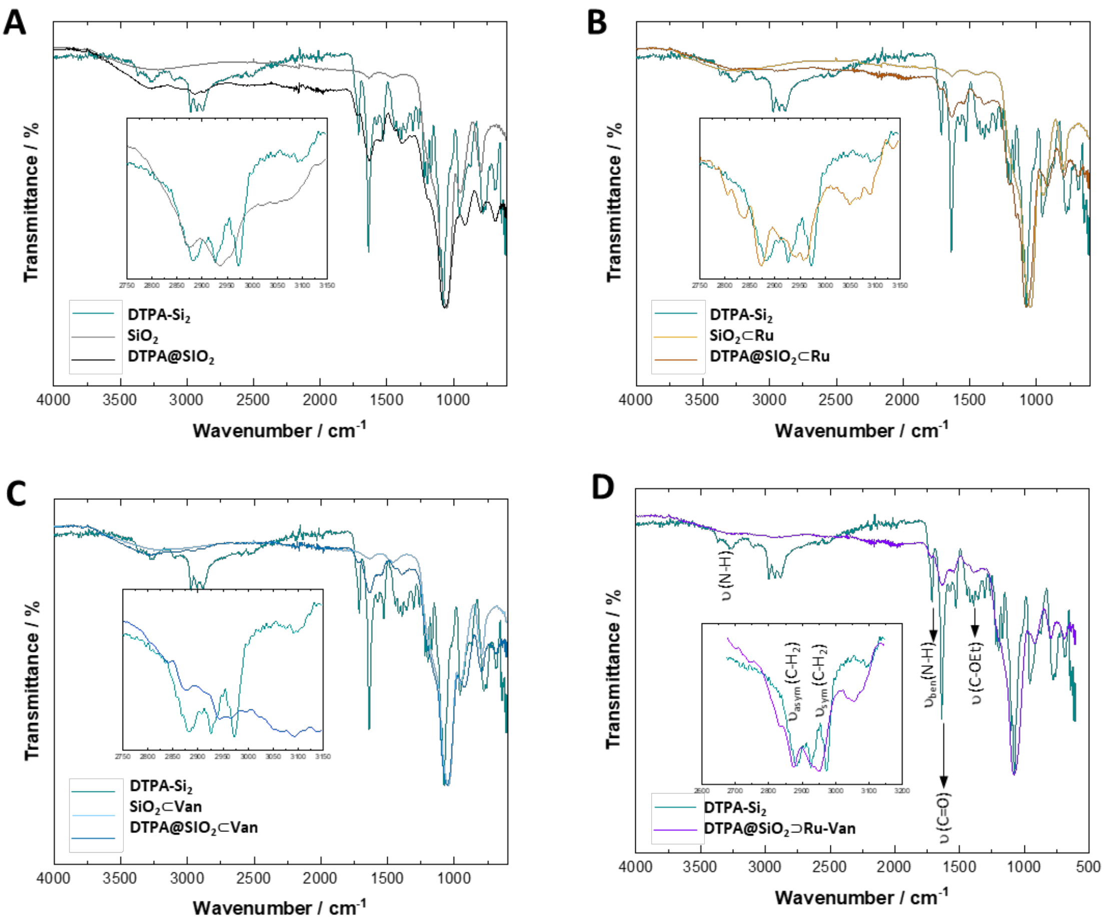

Figure S2. Infrared (IR) spectroscopy spectra of (A) **DTPA@SiO$_2$**, (B) **DTPA@SiO$_2$⊃Ru**, (C) **DTPA@SiO$_2$⊃Van** and (D) **DTPA@SiO$_2$⊃Ru-Van**

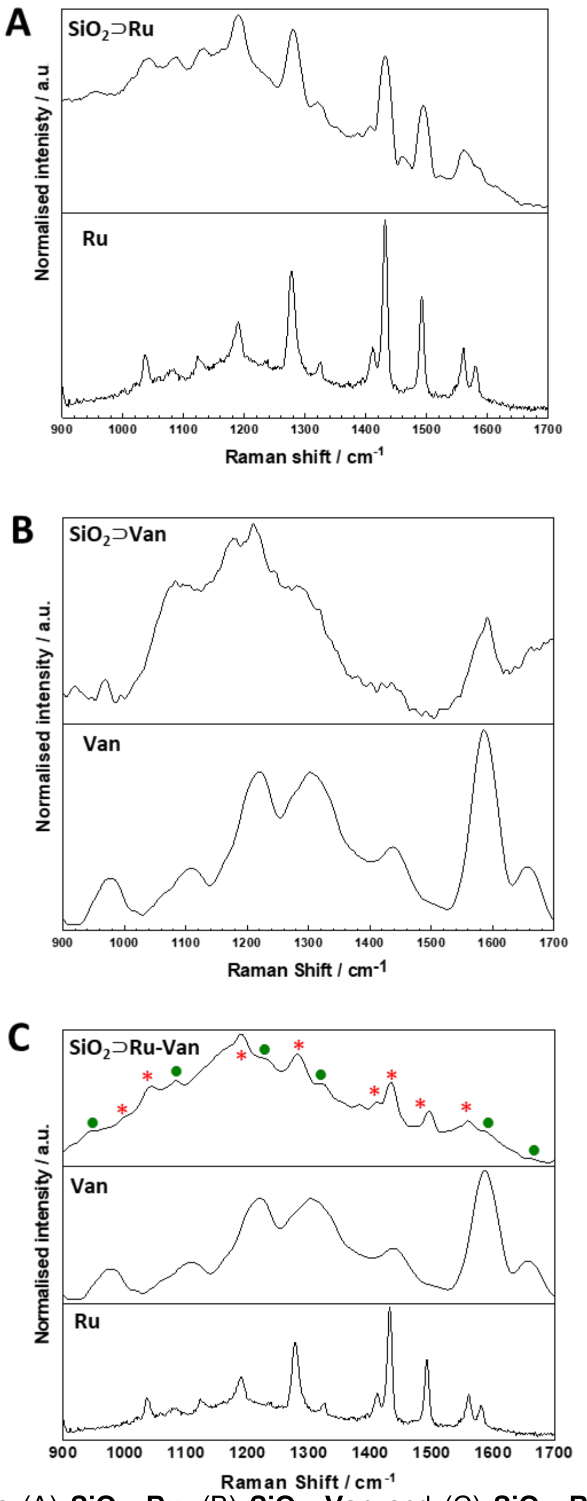

Figure S3. Raman spectra (A) **SiO_2⊃Ru**, (B) **SiO_2⊃Van** and (C) **SiO_2⊃Ru-Van**. Indicates Raman shifts assigned to **Ru** (*) and to **Van** (●) based on comparative spectra.

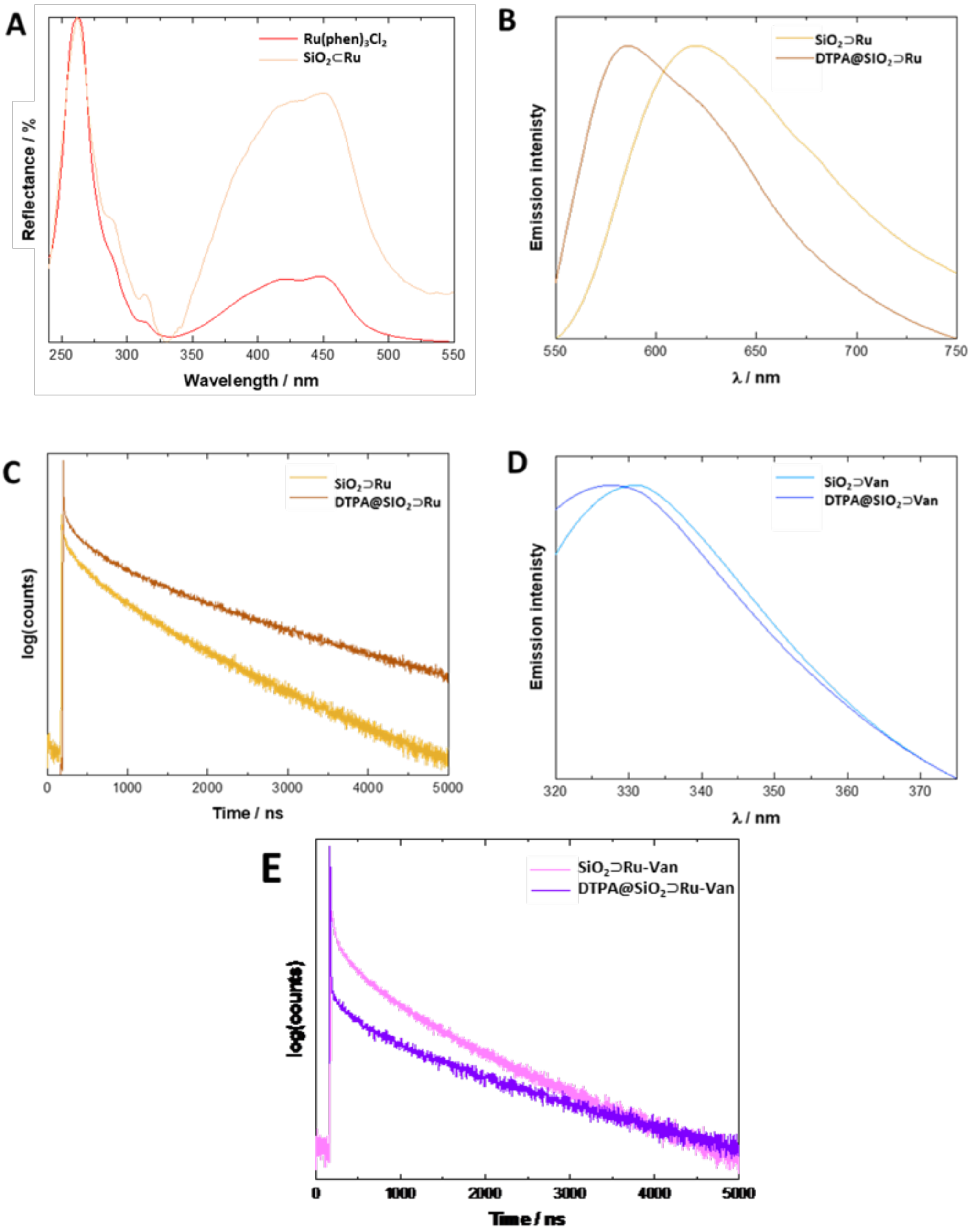

Figure S4. (A) Reflectance UV/Vis spectra in solid-state, (B, D) luminescence spectra (λ_{exc}= 450 for **Ru** and 270 nm for Van) in solid state and exponential lifetime decay (λ_{exc}= 445 nm, λ_{em}= 600 nm) of (C) **SiO_2⊃Ru** and (E) **SiO_2⊃Ru-Van** before and after coating .

Table S3. Solid state photophysical parameters of **Ru complex in solid state** and **Ru-**containing SiO_2 nanoparticles in solid state**.**

	λ_{max} / nm	τ_1 / μs (%)	τ_2 / μs (%)	χ^2
**Ru**	595	1.1 (100)[a]	-	-
**SiO_2⊃Ru**	612	1.45 (89)	0.38 (11)	1.019
**DTPA@SiO_2⊃Ru**	593	3.31 (90)	0.84 (10)	1.004
**SiO_2⊃Ru-Van**	613	1.69 (90)	0.33 (10)	1.021
**DTPA@SiO_2⊃Ru-Van**	595	2.52 (91)	0.34 (9)	1.049

[a] Reported from reference (2) for comparison.

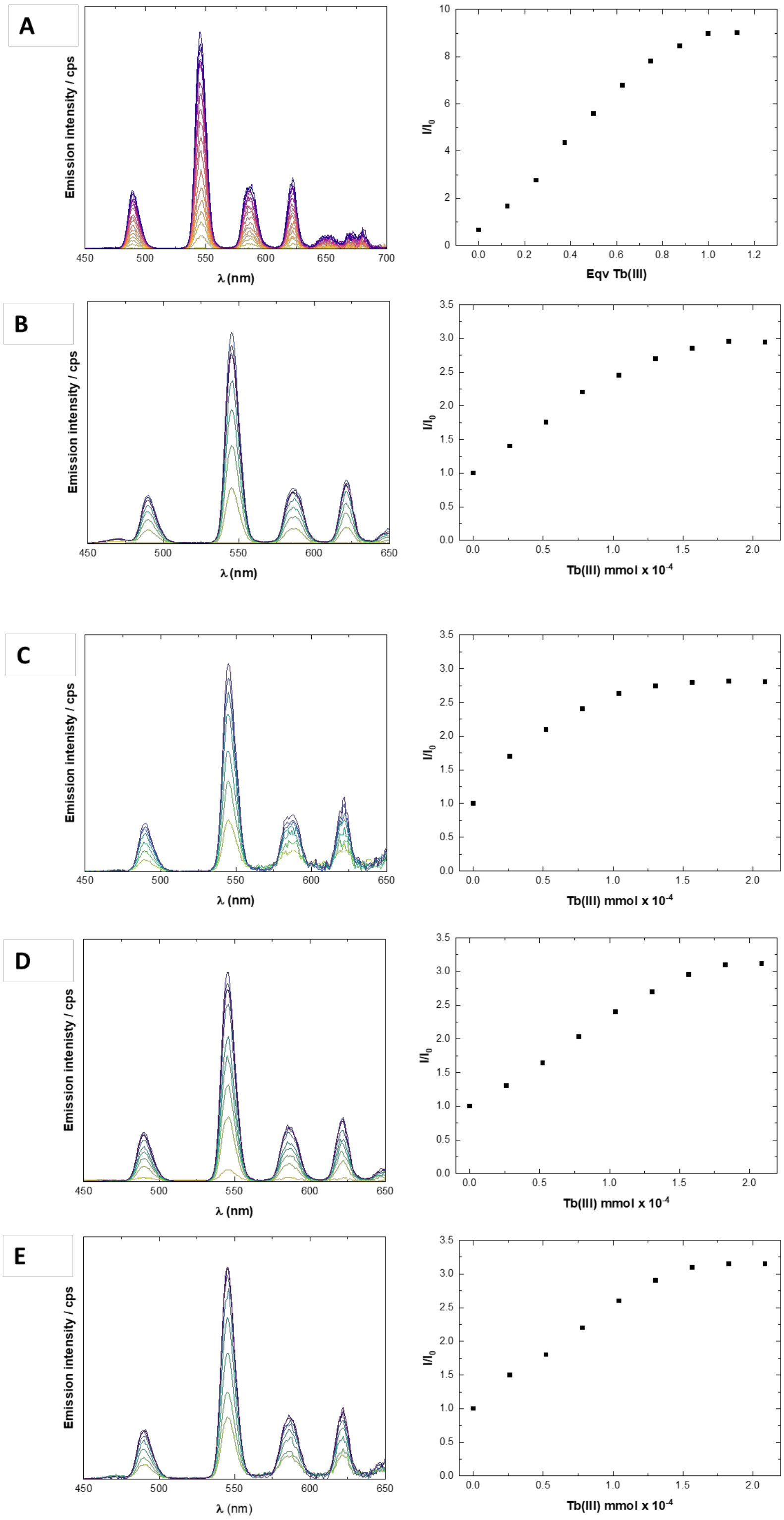

Figure S5. Recorded emission spectra (λ_{exc} = 273 nm) and titration curves of (A) silyl-modified aminocarboxylate DTPA ligand in methanol (4 mM), (B) **DTPA@SiO$_2$**, (C) **DTPA@SiO$_2$⊃Ru**, (D) **DTPA@SiO$_2$⊃Van** and (E) **DTPA@SiO$_2$⊃Ru-Van**. Nanoparticles titrations were performed using a 1 mg/mL methanolic suspension and a methanol Tb(III) solution of 3.3 mM.

Table S4. Loading and DTPA content of nanoparticles.

	Loading / μg.mg_{NP}^{-1}		DTPA /mg.mg_{NP}^{-1}	
	**Uv-Vis**	**TGA**	**TGA**	**Tb(III) titration**
**DTPA@SiO_2**	N/A	N/A	0.55	0.50
**DTPA@SiO_2⊃Ru**	25	26	0.50	0.54
**DTPA@SiO_2⊃ Van**	92	89	0.49	0.53

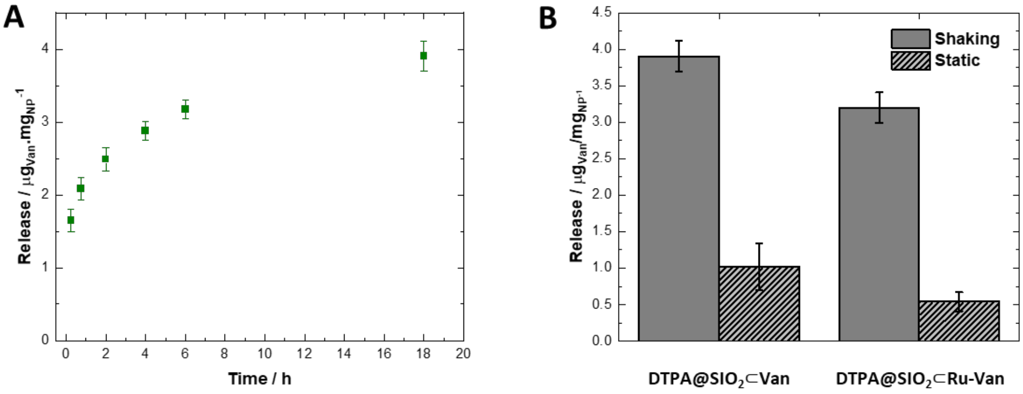

Figure S6. Van release profile from **DTPA@SiO$_2$⊃Van** under shaking at 37 ºC and 150 rpm in water. (B) Quantitative comparative study of released **Van** from **DTPA@SiO$_2$⊃Van** and **DTPA@SiO$_2$⊃Ru-Van** after 90 min of continuous release under shaking (150 rpm) or static conditions. Release was quantified by fluorescence spectroscopy (λ_{exc} = 270 nm and λ_{em} = 330 nm). n = 3

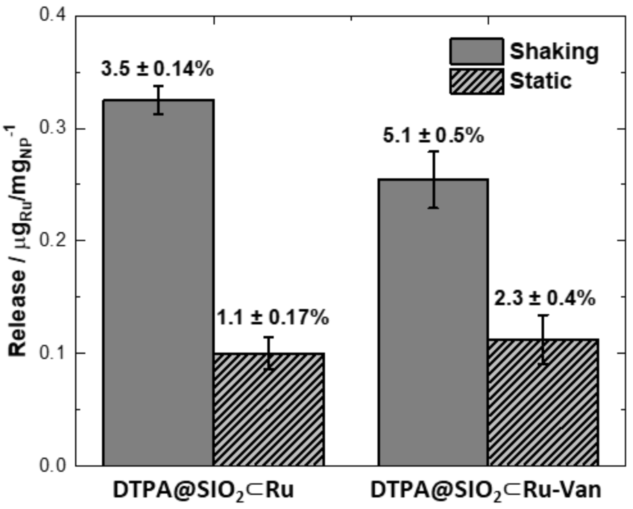

Figure S7. Ru leakage from **DTPA@SiO$_2$⊃Ru** and **DTPA@SiO$_2$⊃Ru-Van** under shaking (150 rpm) and static conditions at 37 °C and in water. Depicted releases were calculated after 120 min and at 37 °C by fluorescence spectroscopy (λ_{exc} = 450 nm and λ_{em} = 600 nm for Ru). n = 3

Table S5. Minimal inhibitory concentration (MIC, mg·mL-1) of $Ru(phen)_3Cl_2$ and Vancomycin HCl active compounds and synthetized nanoparticle library for *S. aureus* (ATTC23851), *E. coli* (MG1665), *A. baumannii* (AC05), *K. pneumoniae* (ATCC43816R), *K. oxytoca* (080-KPC CHD), *C. feundii* (NCTC9750) and *S. enterica* (SL1344) determined by the 2-fold dilution method upon shaking at 150 rpm (black) and under static conditions (red) at 37 °C in LB media. Depicted results are based on 3 biological replicas.

	S. aureus	*E. coli*	*A. baumannii*	*K. pneumoniae*	*K. oxytoca*	*C. freundii*	*S. enterica*
**$Ru(phen)_3Cl_2$**	0.32	1.25	>2.50	2.50	>2.50	>2.50	2.50
	N/A	N/A	N/A	N/A	N/A	N/A	N/A
**Vancomycin HCl**	0.001	>0.250	>0.250	>0.250	>0.250	>0.250	>0.250
	N/A	N/A	N/A	N/A	N/A	N/A	N/A
**SiO_2**	> 10	> 10	> 10	> 10	> 10	> 10	> 10
	> 10	> 10	> 10	> 10	> 10	> 10	> 10
**DTPA@SiO_2**	2.50	> 10	10	10	10	10	10
	2.50	> 10	10	10	10	10	10
**SiO_2⊃Ru**	5.00	>10	>10	>10	>10	>10	>10
	1.25	>10	>10	>10	>10	>10	>10
**DTPA@SiO_2⊃Ru**	1.25	>10	>10	>10	>10	>10	>10
	0.75	5.00	5.00	1.25	5.00	10	5.00
**SiO_2⊃Van**	0.75	5.00	10	5.00	10	5.00	10
	0.75	5.00	10	10	10	5.00	10
**DTPA@SiO_2⊃Van**	0.37	5.00	5.00	10	5.00	5.00	5.00
	0.37	2.50	2.50	2.50	1.25	1.25	2.50
**SiO_2⊃Ru-Van**	2.50	10	10	>10	10	10	10
	1.25	5.00	2.50	2.50	5.00	5.00	2.50
**DTPA@SiO_2⊃Ru-Van**	0.37	5.00	5.00	5.00	10	10	5.00
	0.37	1.25	1.25	0.75	1.25	1.25	0.75

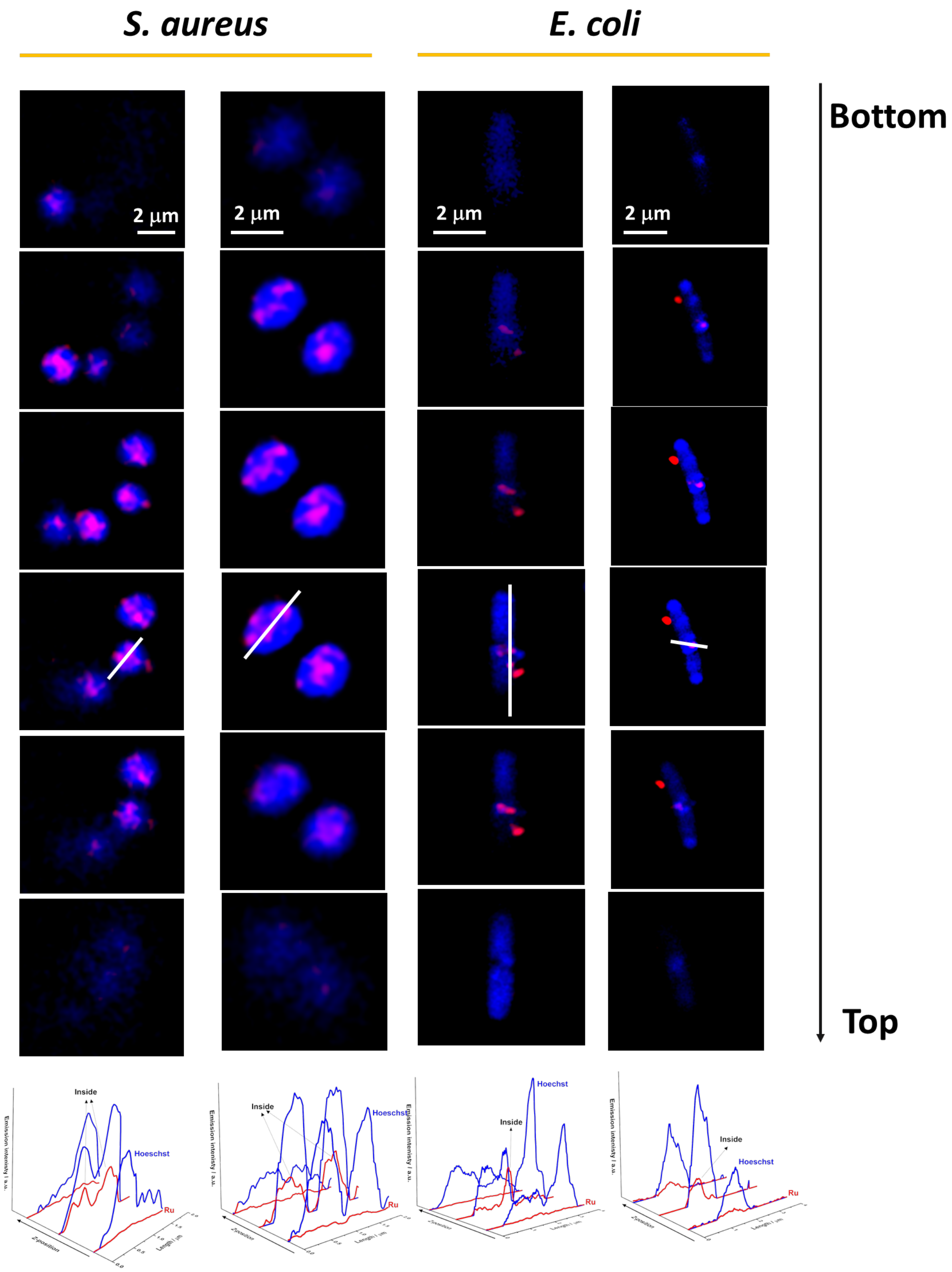

Figure S8. Emission profiles generated from z-stack analysis (Figure 4) for selected stained *S. aureus* and *E. coli* cells incubated with **DTPA@SiO$_2$⊃Ru-Van** with a final nanoparticle concentration of 2.5 mg/mL for 2 h at 37 °C under mild shaking (150 rpm). Red channel, Ru emission (λ_{exc} = 488 nm, λ_{em} = 560 – 620 nm); blue channel Hoechst H33342 emission (λ_{exc} = 405 nm, λ_{em} = 420 – 450 nm).

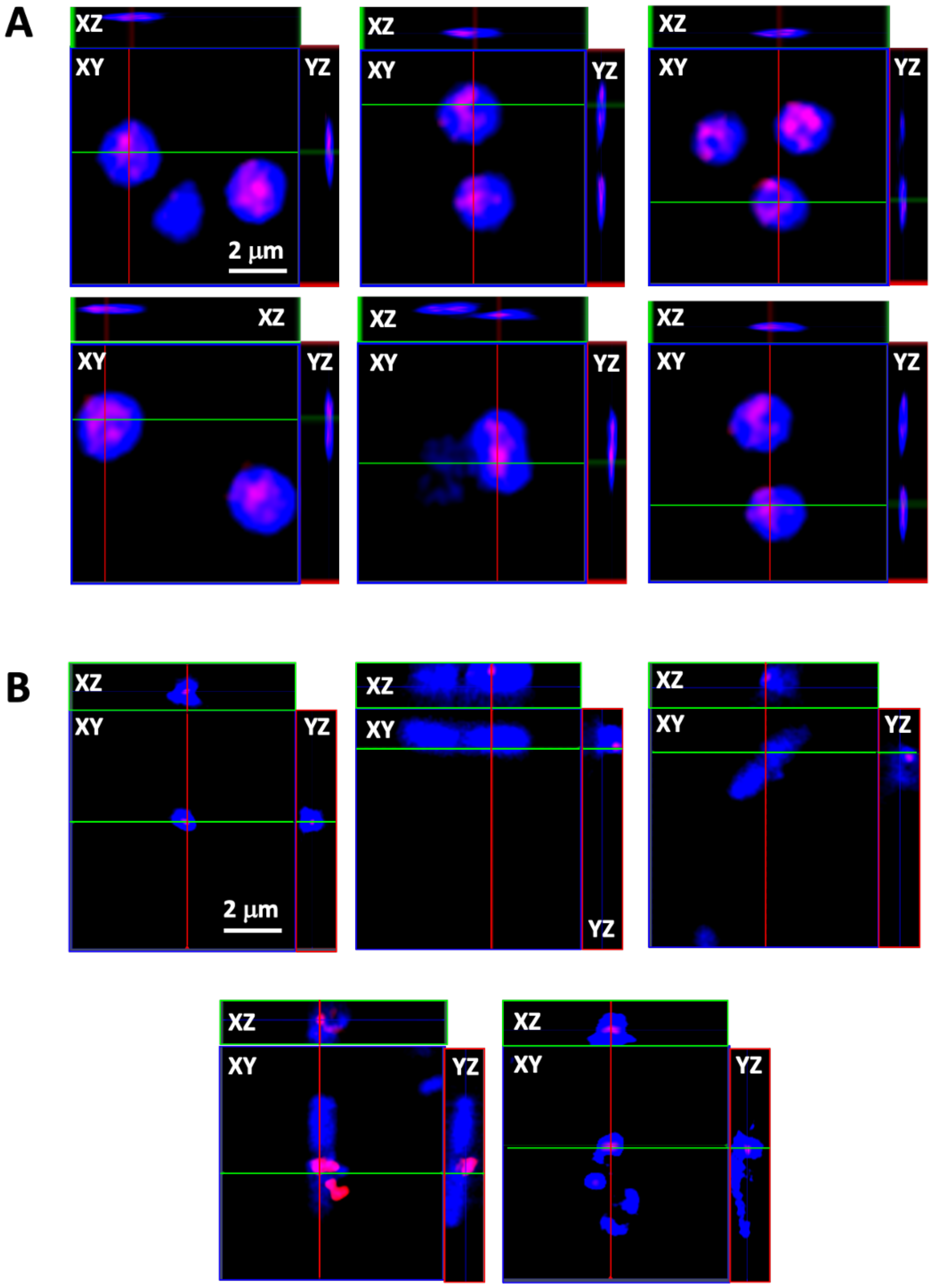

Figure S9. Orthogonal reconstruction of selected central plane from z-stack images of further biological replicates from *S. aureus* (A) and *E.coli* (B) cells showing ruthenium signal inside cells after incubation with **DTPA@SiO$_2$⊃Ru-Van**. Overnight grown bacteria were incubated with **DTPA@SiO$_2$⊂Ru-Van** with a final nanoparticle concentration of 2.5 mg/mL for 2 h at 37 °C under mild shaking (150 rpm). Red channel, ruthenium emission (λ_{exc} = 488 nm, λ_{em} = 560 – 620 nm); blue channel Hoechst H33342 emission (λ_{exc} = 405 nm, λ_{em} = 420 – 450 nm).

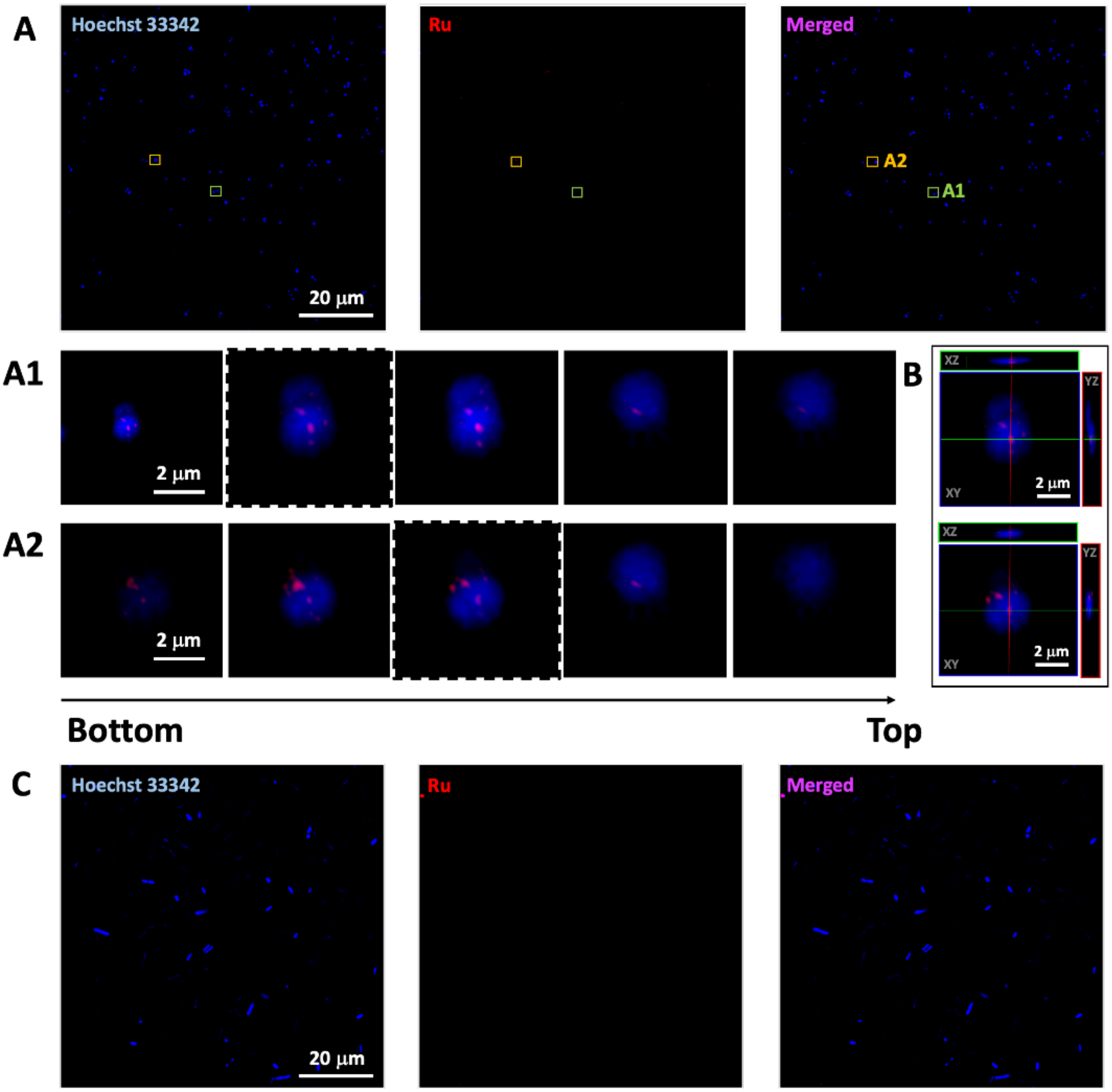

Figure S10. Structural illumination microscopy (SIM) imaging of uncoated particles, **SiO$_2$⊂Ru-Van.** Representative live cell (n = 5) images *S. aureus* (A, A1 and A2) and *E. coli* (C) after incubation with **SiO$_2$⊃Ru-Van**. Overnight grown bacteria were incubated with a final nanoparticle concentration of 2.5 mg/mL for 2 h at 37 °C under mild shaking (150 rpm). Red channel, ruthenium emission (λ_{exc} = 405 nm, λ_{em} = 420 – 450 nm); blue channel Hoechst H33342 emission (λ_{exc} = 488 nm, λ_{em} = 560 – 620 nm). Orthogonal reconstruction (B) of central plane from z-stack (highlighted with dashed line) of *S. aureus* cells containing **SiO$_2$⊃Ru-Van.**

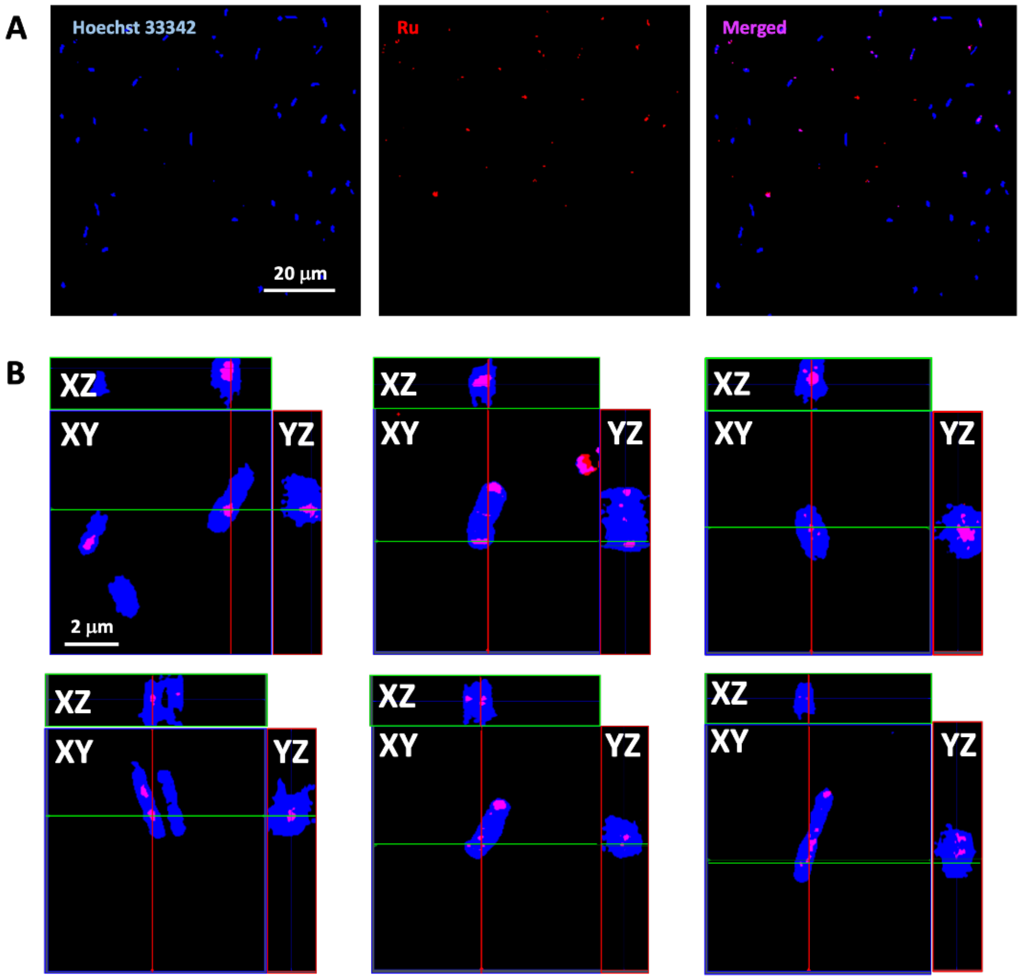

Figure S11. Live cell structural illumination microcopy of bacteria cells with 18h incubation time of **DTPA@SiO$_2$⊃Ru-Van** in *E. coli* (n = 5). Images show each channel for the outline of bacteria cells (blue) and particle luminescence (red) as well as merged channels (A) and orthogonal reconstruction of 3D z-stack images (B). Overnight grown bacteria were incubated with **DTPA@SiO$_2$⊂Ru-Van** with a final nanoparticle concentration of 2.5 mg/mL at 37 °C under mild shaking (150 rpm). Red channel, ruthenium emission (λ_{exc} = 488 nm, λ_{em} = 560 – 620 nm); blue channel Hoechst H33342 emission (λ_{exc} = 405 nm, λ_{em} = 420 – 450 nm).

Table S6. Colocalization between blue and red signal in E. coli cells was analysed by the Fiji plug in JACoP (3) calculating the Pearson and Manders' Overlap coefficient (n = 5) after different incubation times. M1 corresponds to the relative blue signal (Hoechst, bacterium cell) overlapping with the red channel (ruthenium, nanoparticles). M2 corresponds to the relative red signal overlapping with the blue one.

		JACoP		
		**Pearson analysis**	**Manders' Overlap coefficient**	
	**Incubation**	**r**	**M1**	**M2**
**SiO_2⊃Ru-Van**	2 h	0.42 ± 0.10	0.03 ± 0.02	0.10 ± 0.04
	18 h	0.47 ± 0.05	0.08 ± 0.05	0.20 ± 0.07
**DTPA@SiO_2⊃Ru-Van**	2 h	0.48 ± 0.16	0.55 ± 0.16	0.61 ± 0.13
	18 h	0.52 ± 0.13	0.75 ± 0.11	0.87 ± 0.07

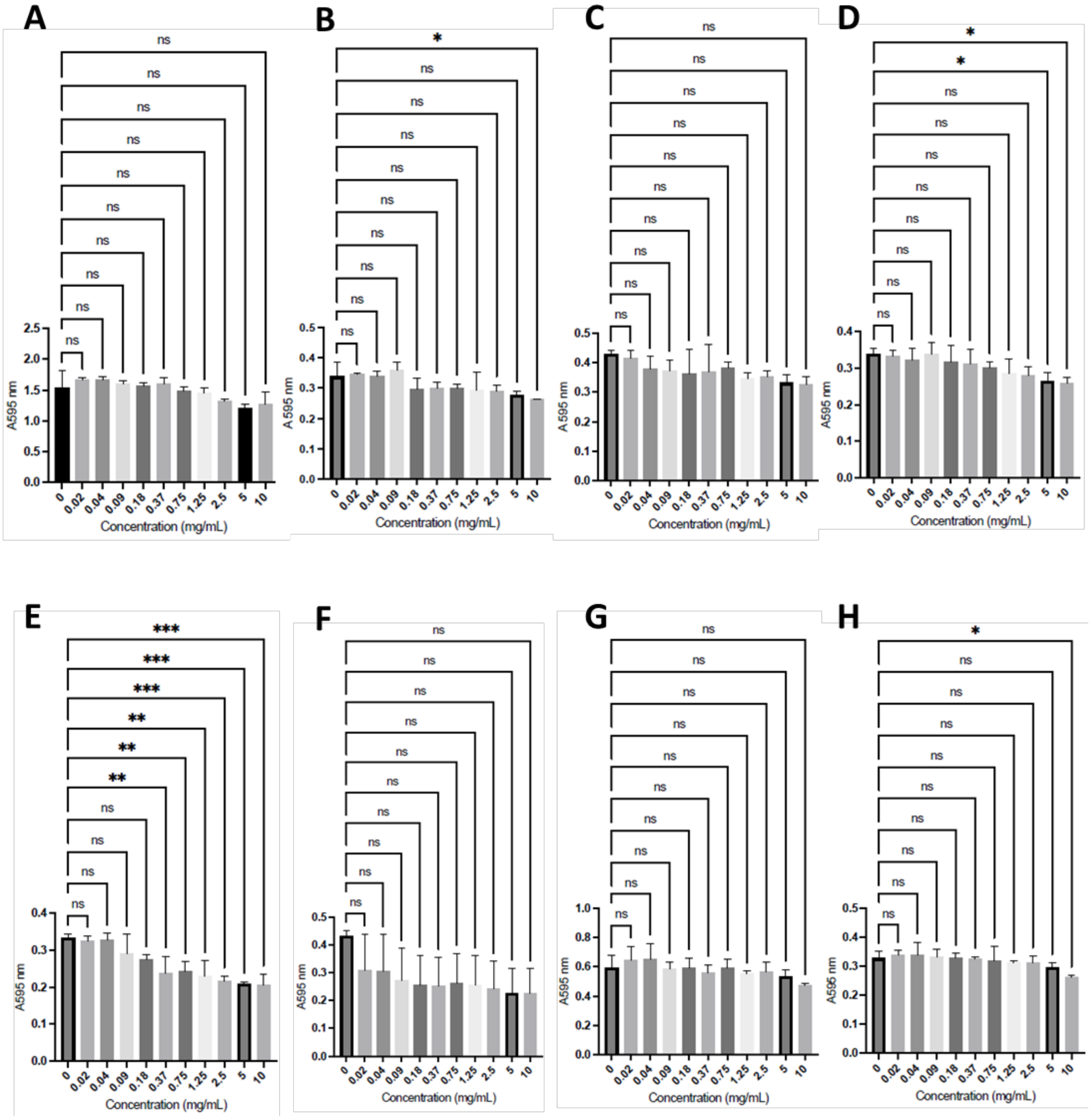

Figure S12. MTT cell viability assays of A549 cell line incubated with studied nanoparticles for 18 hours with (A) **SiO_2**, (B) **DTPA@SiO_2**, (C) **SiO_2⊃Ru**, (D) **DTPA@SiO_2⊃Ru**, (E) **SiO_2⊃Van,** (F) **DTPA@SiO_2⊃Van** (G) **SiO_2⊃Ru-Van** and (H) **DTPA@SiO_2⊃Ru-Van** 1-way ANOVA followed by a Dunnett's t-test corrected for multiple comparisons was performed, * = $P<0.05$, ** = $P<0.01$ and *** = $P< 0.001$ n = 3.

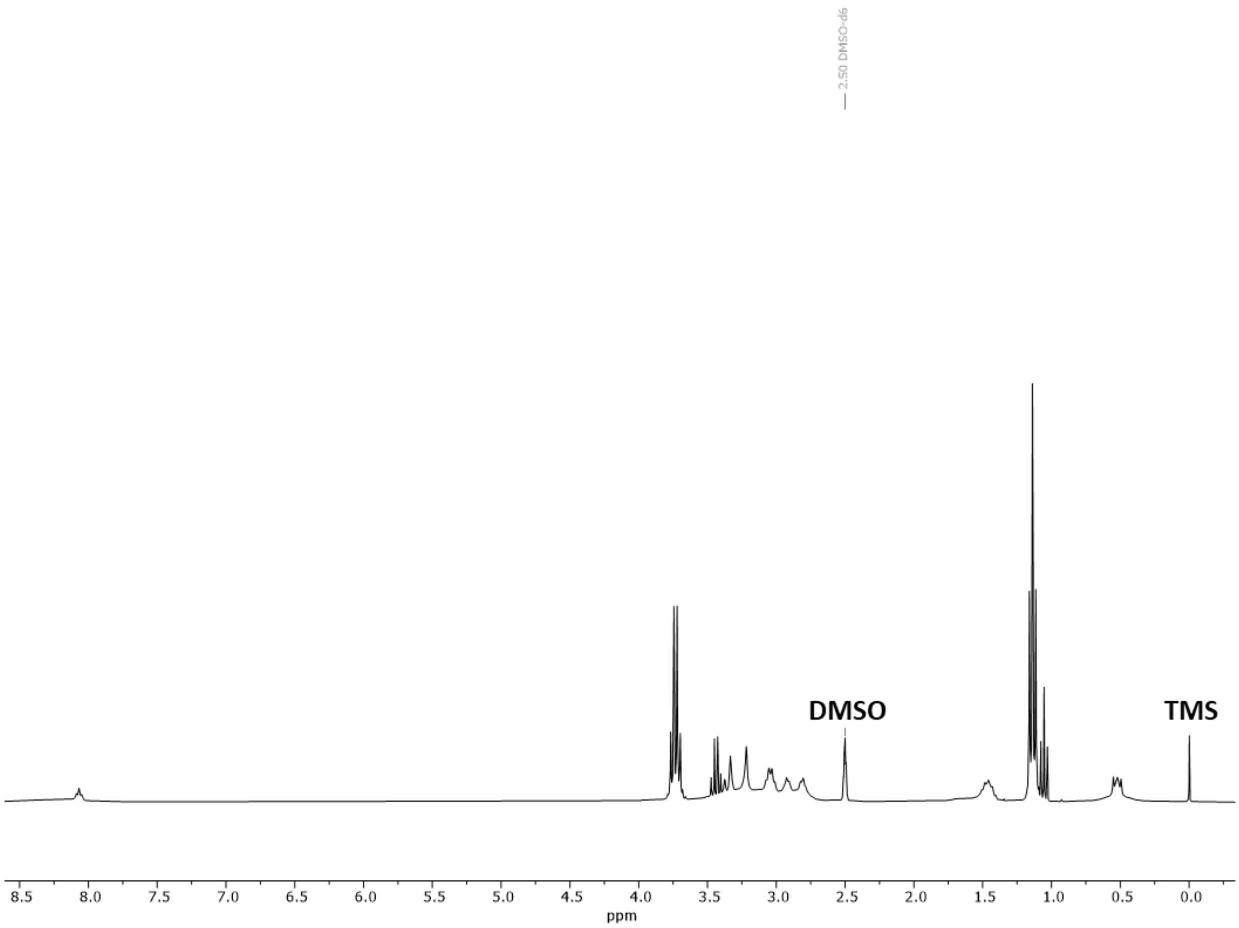

Figure S13. ^{1}H-NMR (400 MHz, d_6-DMSO) spectrum of DTPA-Si_2.

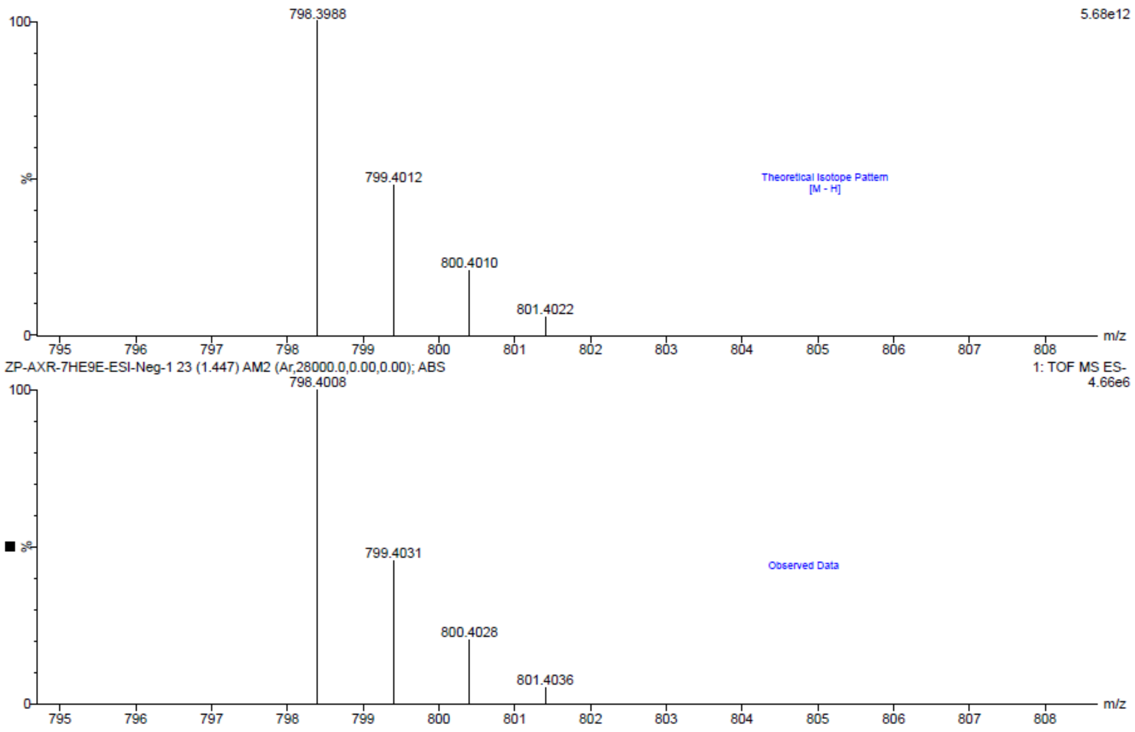

Figure S14. Mass spectra ESI-ToF (-) of DTPA-Si_2.

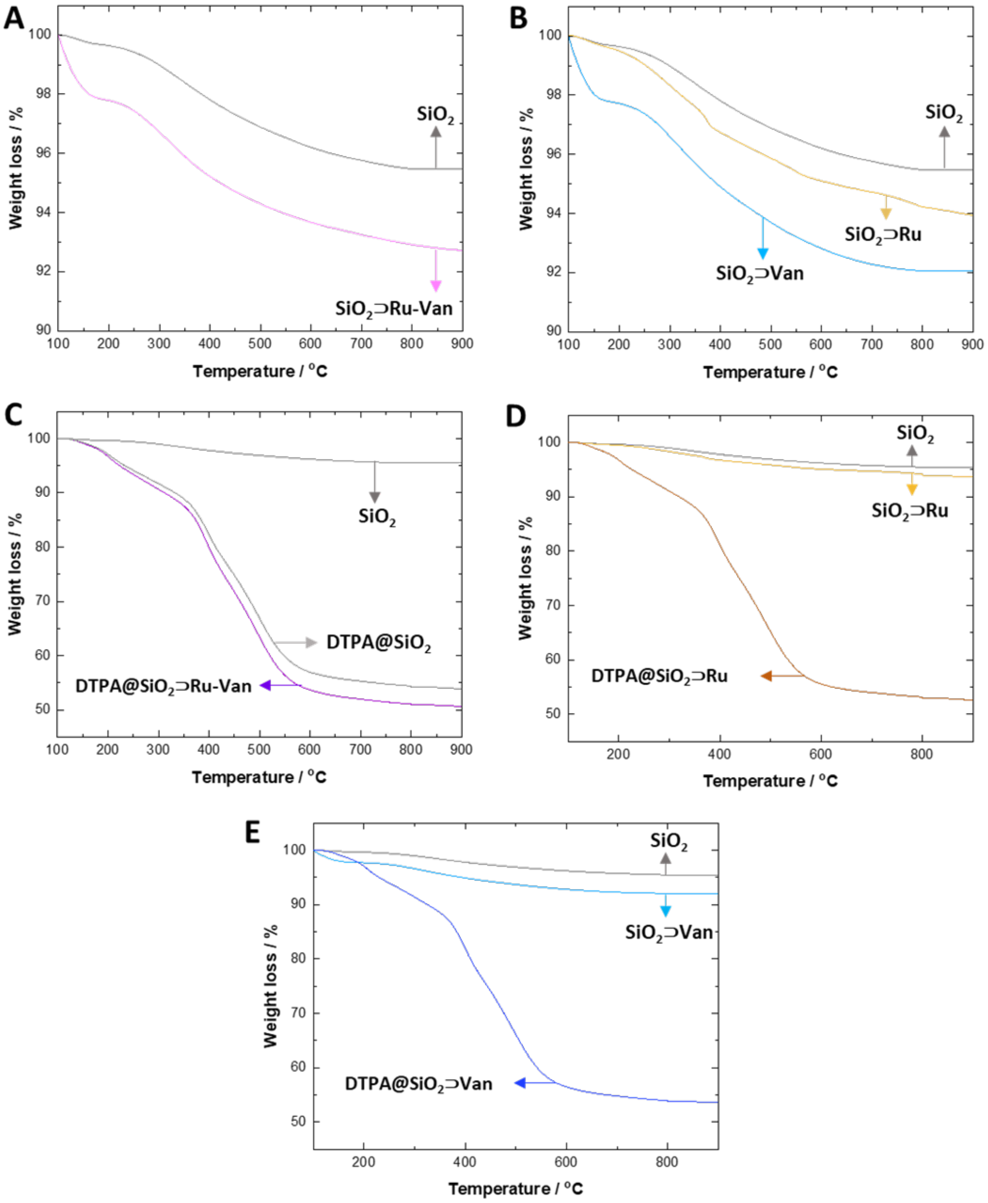

Figure S15. Quantification of included cargo in (A) **SiO_2⊃Ru-Van** and (B) **SiO_2⊃Ru** and **SiO_2⊃Van** and DTPA quantification for (C) **DTPA@SiO_2⊃Ru-Van,** (D) **DTPA@SiO_2⊃Ru** and (E) **DTPA@SiO_2⊃Van** Samples are heated from 30 ºC to 900 ºC at 1 ºC /min rate with a 10 min isothermal step at 100 ºC to ensure water removal. All degradation curves are set at 100 % for clarity.

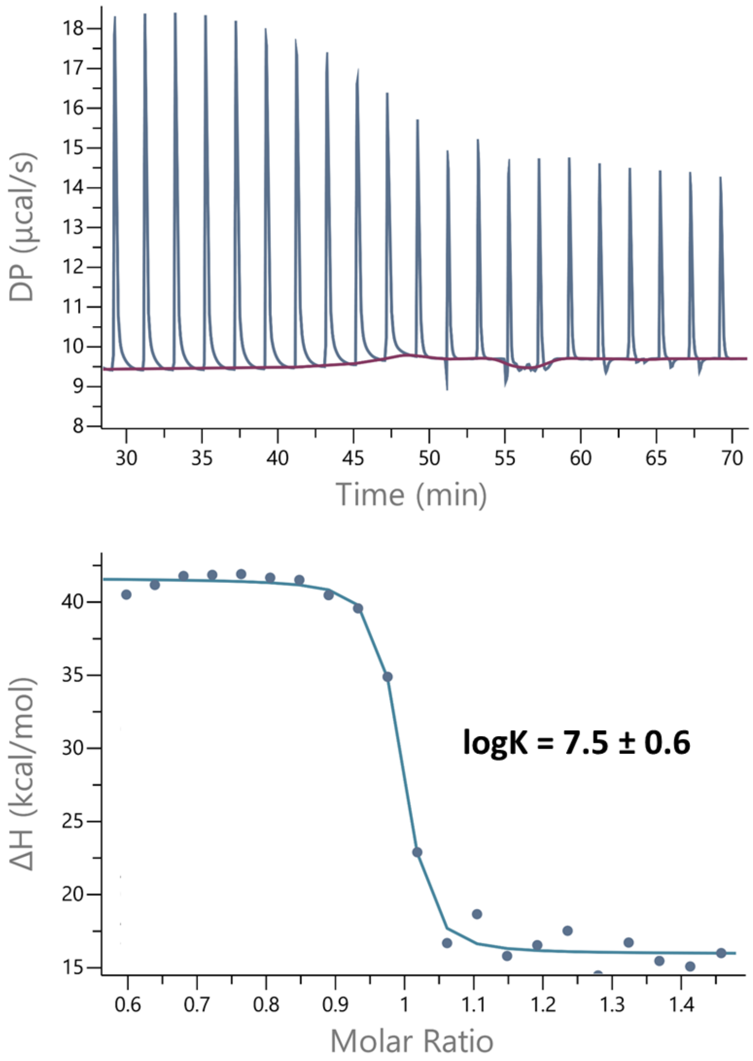

Figure S16. Isothermal Titration Calorimetry (ITC) graph and fitted data of Ca^{2+} (2.5 mM) into a suspension of **DTPA@SiO$_2$⊃Ru-Van** ([DTPA] = 0.32 mM) in PBS (0.1 M, pH = 7.4) at 25 °C.

Table S7. Minimal inhibitory concentration (MIC, mg·mL-1) of **SiO_2⊃Ru-Van**, **DTPA@SiO_2⊃Ru-Van** and **Ca^{2+}DTPA@SiO_2⊃Ru-Van** for *S. aureus* (ATTC23851), *E. coli* (MG1665), *A. baumannii* (AC05), *K. pneumoniae* (ATCC43816R), *K. oxytoca* (080-KPC CHD), *C. feundii* (NCTC9750) and *S. enterica* (SL1344) determined by the 2-fold dilution method upon shaking at 150 rpm (black) and under static conditions (red) at 37 °C in LB media (n=3).

	S. aureus	*E. coli*	*A. baumannii*	*K. pneumoniae*	*K. oxytoca*	*C. freundii*	*S. enterica*
**SiO_2⊃Ru-Van**	2.50	10	10	>10	10	10	10
	1.25	5.00	2.50	2.50	5.00	5.00	2.50
**DTPA@SiO_2⊃Ru-Van**	0.37	5.00	5.00	5.00	10	10	5.00
	0.37	1.25	1.25	0.75	1.25	1.25	0.75
**Ca^{2+}DTPA@SiO_2⊃Ru-Van**	2.50	>10	10	>10	10	10	10
	2.50	5.00	10	5.00	5.00	5.00	5.00

References

(1)D. J. Lewis, V. Sore, N. J. Rogers, T. K. Mole, G. B, Nash, P. Angeli, Z. Pikramenou. *Langmuir,* **2013**, 29, 14701-14709.

(2)N. Kobayashi, H. Minami, K. Nakamura. *Nanophotonics*, **2018**, **7**, 1373-1385.

(3)S. Bolte & F. P. Cordelieres, *J. Microsc*., **2006**, 224, 2013-232.
